# Holotranscobalamin (HoloTC, Active-B12) and Herbert’s model for the development of vitamin B_12_ deficiency: a review and alternative hypothesis

**DOI:** 10.1186/s40064-016-2252-z

**Published:** 2016-05-20

**Authors:** Paul Henry Golding

**Affiliations:** Unit 5, 18 Webster Road, Nambour, QLD 4560 Australia

**Keywords:** Holotranscobalamin, HoloTC, Active-B12, Vitamin B_12_, Methylmalonic acid

## Abstract

The concentration of total vitamin B_12_ in serum is not a sufficiently sensitive or specific indicator for the reliable diagnosis of vitamin B_12_ deficiency. Victor Herbert proposed a model for the staged development of vitamin B_12_ deficiency, in which holotranscobalamin (HoloTC) is the first indicator of deficiency. Based on this model, a commercial immunoassay has been controversially promoted as a replacement for the total vitamin B_12_ test. HoloTC is cobalamin (vitamin B_12_) attached to the transport protein transcobalamin, in the serum, for delivery to cells for metabolism. Although there have been many published reports supporting the claims for HoloTC, the results of some studies were inconsistent with the claim of HoloTC as the most sensitive marker of vitamin B_12_ deficiency. This review examines the evidence for and against the use of HoloTC, and concludes that the HoloTC immunoassay cannot be used to measure vitamin B_12_ status any more reliably than total vitamin B_12_, or to predict the onset of a metabolic deficiency, because it is based on an erroneous hypothesis and a flawed model for the staged development of vitamin B_12_ deficiency. The author proposes an alternative model for the development of vitamin B_12_ deficiency.

**Electronic supplementary material:**

The online version of this article (doi:10.1186/s40064-016-2252-z) contains supplementary material, which is available to authorized users.

## Background

### Herbert’s model

In his Herman Award Lecture of 1986, Victor Herbert proposed his *Sequential stages in the development of vitamin B*_12_*deficiency* (Herbert 1987). Herbert’s hypothesis, describing the *Biochemical and hematological sequence of events as negative vitamin B*_12_*balance progresses*, two decades later formed the basis for the introduction of the commercial holotranscobalamin (HoloTC, Active B12) immunoassay for the diagnosis of vitamin B_12_ deficiency.

The concentration of total vitamin B_12_ in serum is not a sufficiently sensitive or specific indicator for the reliable diagnosis of vitamin B_12_ deficiency (Herbert 1987, 1994; Hølleland et al. 1999; Snow 1999; Oh and Brown 2003; Solomon 2005; Herrmann and Obeid 2008, 2013; Schrempf et al. 2011; Heil et al. 2012). Herbert observed that, as with iron and folate, the range of normal concentrations of serum total vitamin B_12_ for an individual is narrower than the range of normal for a population (Herbert 1987): “the laboratory test result becomes abnormal for the individual before it exceeds the range of normal for the laboratory”. As observed by Snow “As knowledge has accumulated, the limitations of such tests as serum vitamin levels … have become apparent”.

The two metabolites of vitamin B_12_, homocysteine (tHcy) and methylmalonic acid (MMA) have been proposed as more sensitive than total serum vitamin B_12_, for the diagnosis of vitamin B_12_ deficiency (Hølleland et al. 1999; Snow 1999; Oh and Brown 2003). In support of the use of these metabolites Oh and Brown say that “use of a low serum vitamin B_12_ level as the sole means of diagnosis may miss up to one half of patients with actual tissue B_12_ deficiency”. In advocating the use of methylmalonic acid, Hølleland et al. state that “Our data emphasize the poor diagnostic utility of low and low-normal s-cobalamin assay results and call for more sensitive and specific markers of cobalamin deficiency. s-MMA meets these criteria”. Solomon (2005) differed, finding that methylmalonic acid and homocysteine were also unreliable for the diagnosis of vitamin B_12_ deficiency.

Even now, after decades of research, there is still no agreed *gold*-*standard* test for vitamin B_12_ deficiency. As recently stated by Aparicio-Ugarriza et al. (2014): “There is no consensus in the literature about cut-off points for blood vitamin B_12_ reference ranges and its associated metabolites.” It is against the background of this unmet need that the holotranscobalamin (HoloTC, Active B12) immunoassay has been offered as a solution.

Holotranscobalamin (HoloTC) is cobalamin (vitamin B_12_) attached to the transport protein transcobalamin (TC), in the serum, for delivery to cells for metabolism. The absorption and transport of vitamin B_12_ is described in detail in Neale (1990). Recent developments in understanding the role of HoloTC in the assimilation and metabolism of vitamin B_12_ are explained in detail by Quadros (2010). Fedosov (2010, 2013) has produced a mathematical model for the biochemical markers of vitamin B_12_ deficiency, including HoloTC.

Herbert proposed serum holotranscobalamin (HoloTC) as an earlier and more sensitive indicator of vitamin B_12_ deficiency than total vitamin B_12_ (Herbert 1987, 1994). According to Herbert (1994): “Low concentrations of holoTCII occur before low concentrations of total serum vitamin B-12 or before deficiency”. This is based on two properties of HoloTC; the small percentage of total vitamin B_12_ existing as HoloTC, and the short half-life of HoloTC. According to Herbert (1994), HoloTC comprises only 20 % of serum total vitamin B_12_; this metabolically active portion is carried to all cells on the transport protein transcobalamin (TC). The remaining 80 % (holohaptocorrin, HoloHC) is the metabolically inert component carried on the liver storage protein haptocorrin (HC). According to Herbert (1994), HoloTC has a short half-life of only 6 min compared to 240 h for HoloHC. There is disagreement about the actual half-life of HoloTC, as discussed later in this review, but the consensus is that it is far shorter than that of HoloHC.

**Fig. 1:**
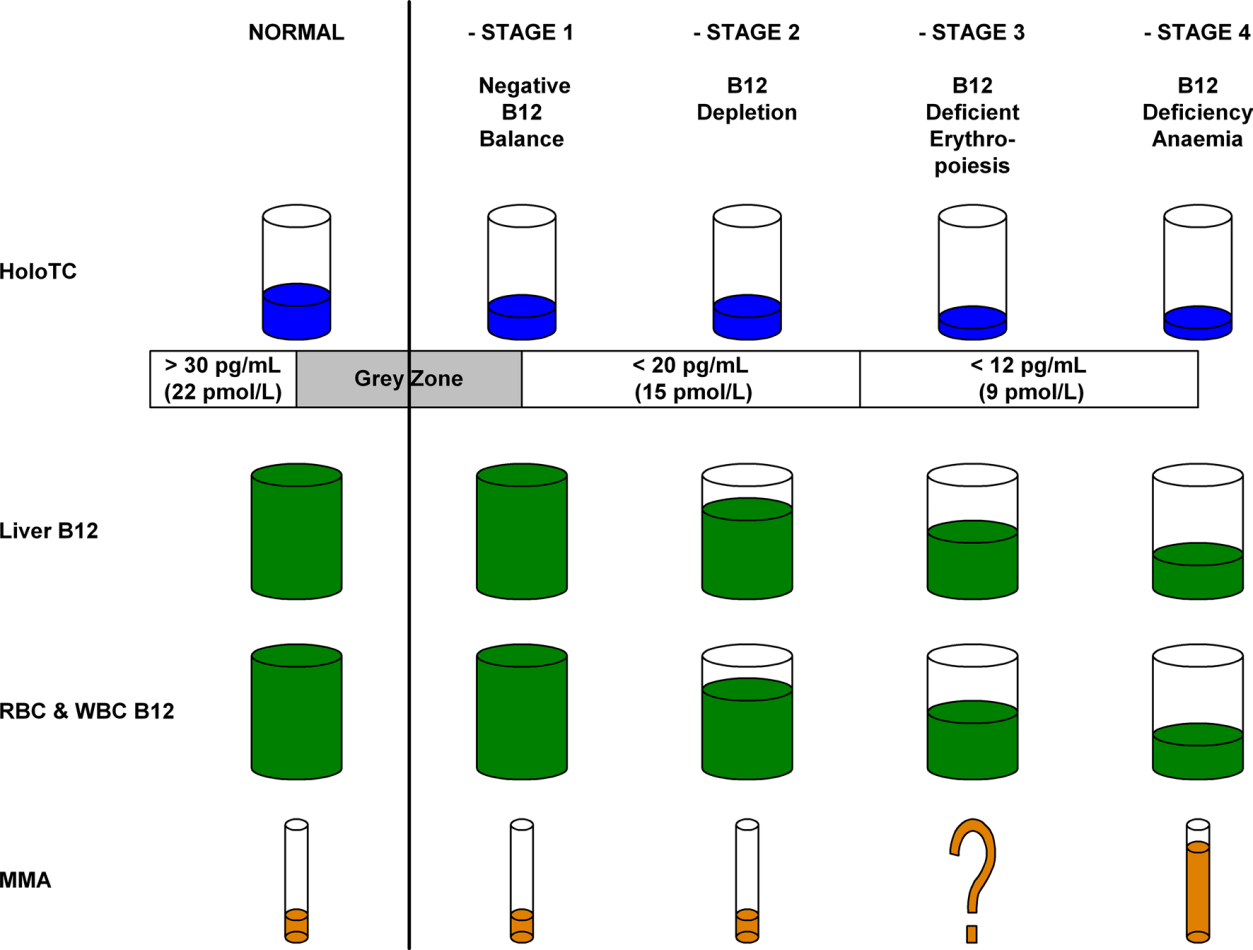
Herbert’s 1986 model for sequential stages in the development of vitamin B_12_ deficiency. This simplified model was derived from Herbert (1987)

According to Herbert’s 1986 model (Herbert 1987), vitamin B_12_ deficiency develops in a sequence of four distinct stages, with changes in specific biochemical and haematological markers defining the borders between them (Fig. 1; Table 1). The earliest sign of vitamin B_12_ deficiency is marked by the change from *Normal* to *Negative B*_12_*Balance*, with HoloTC concentration falling from >22 to <15 pmol/L, and TC saturation falling from >5 to <5 %. Haematology is normal for the first two stages; initial abnormal haematology, in the form of neutrophil hypersegmentation, appears in stage 3; overtly abnormal haematology in the form of neutrophil hypersegmentation, macroovalocytes, elevated MCV and low haemoglobin, appears in stage 4. MMA is possibly increased in stage three and definitely increased in stage four.

**Table 1:** Herbert’s hypothesis—differences between 1986 and 1994 models

Analyte	Model	Deficiency stage				
			1	2	3	4
	1986	Normal	Negative B_12_ balance	B_12_ depletion	B_12_-deficient erythropoiesis	B_12_-deficiency anaemia
	1994	Normal	Early negative B_12_ balance	B_12_ depletion	Damaged metabolism: B_12_-deficient erythropoiesis	Clinical damage: B_12_-deficiency anemia
HoloTC (pmol/L)	1986	>22	<15	<15	<9	<9
HoloTC (pmol/L)	1994	>37	<30	<30	<30	<30
TC saturation (%)	1986	>5	<5	<2	<1	<1
TC saturation (%)	1994	>5	<4	<4	<4	<4
HoloHC (pmol/L)	1986	>111	>111	<111	<74	<74
HoloHC (pmol/L)	1994	>133	>133	<111	<74	<74
MMA high	1986	No	No	No	?	Yes
MMA high	1994	No	No	No	?	Yes
tHcy high	1986	–	–	–	–	–
tHcy high	1994	No	No	No	Yes	Yes

Data in this table was derived from Herbert (1987, 1994)

HoloTC concentration converted from pg/mL to pmol/L, using formula from Herbert (1987): HoloTC (pmol/L) = HoloTC (pg/mL) × 0.74

Two stages preceding “Normal”, added by Herbert in the 1994 model, omitted from this table

From his 1986 model, Herbert concluded that “By measuring holo TC II one can diagnose negative vitamin B-12 balance, a stage which precedes the stage of B-l2 depletion”, and thus proposed HoloTC to be more sensitive than total serum vitamin B_12_ as an indicator of the onset of vitamin B_12_ deficiency. Herzlich and Herbert (1988) stated: “The data suggest low holo TC II indicates negative B_12_ balance early in the development of tissue depletion of B_12_ … We believe that this parameter provides a new tool for assessing nutritional status in equivocal states”. Wickramasinghe and Fida (1993) supported Herbert’s hypothesis, concluding that “Our data support the model of developing B_12_ deficiency proposed by Herbert and his colleagues and their view that a proper study of B_12_ values should include not only measurements of total serum B_12_ but also of holoTCII concentration”. Neale (1990) more cautiously commented on the hypothesis: “A reduction in the circulating concentration of holo-TCII may be the earliest sign of vitamin B_12_ deficiency. This potentially important observation awaits confirmation”.

**Fig. 2:**
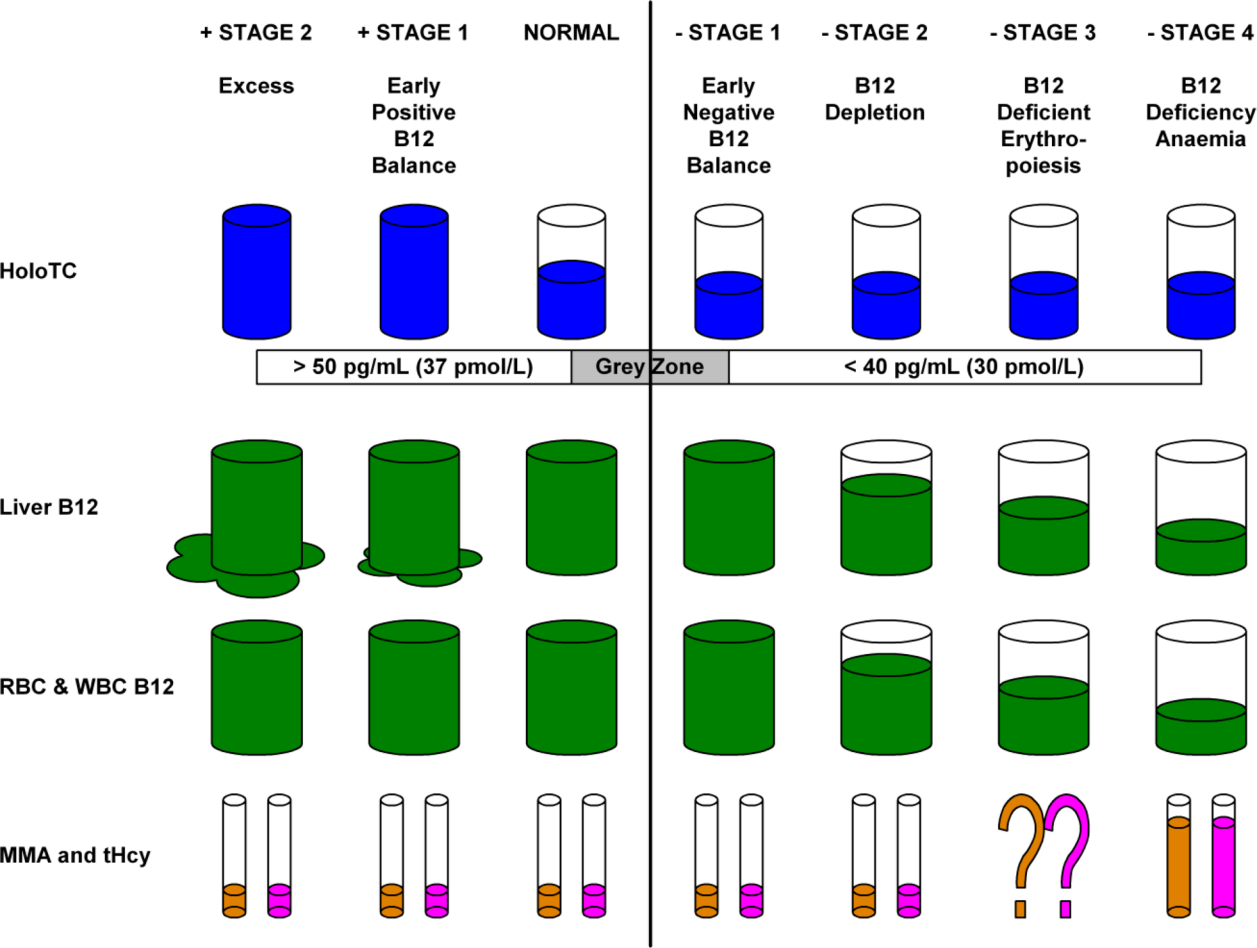
Herbert’s (1994) model for sequential stages in the development of vitamin B_12_ deficiency. This simplified model was derived from Herbert (1994)

Herbert modified his model in 1990 and again in 1994 (Herbert 1994), adding two *Positive Balance* stages before the *Normal* stage, and changing the HoloTC and TC thresholds (Fig. 2; Table 1). Homocysteine, the second metabolite of vitamin B_12_, is included in this revised model. These changes broaden the range of analytes considered while reducing the relative differences in analyte concentrations, particularly HoloTC concentration and TC saturation, between stages. The range of HoloTC concentration thresholds has been reduced significantly, with the difference between normal and deficient conditions much less clear.

The most important effect of the reduction in the range of HoloTC concentration thresholds is in the diagnosis of the very earliest progression from normality to deficiency; the detection of *Negative vitamin B*_12_*Balance* (Table 1). In Herbert’s original 1986 model, HoloTC concentration needs to fall from >22 to <15 pmol/L, to progress from *Normal* to *Negative Vitamin B*_12_*Balance*, a fall of 33 %. In the revised model of 1994, HoloTC concentration needs to fall from >37 to <30 pmol/L, to progress from *Normal* to *Early Negative Vitamin B*_12_*Balance*, a fall of 20 %. Because of the smaller percentage fall in HoloTC required to change from normal to abnormal in the revised model, despite the absolute value of the differences between these stages remaining the same, the diagnosis of *Negative Vitamin B*_12_*Balance* now requires greater precision and accuracy in the individual assay and greater certainty of the HoloTC cut-off levels.

### Commercialisation of the HoloTC immunoassay

Commercialisation of the HoloTC test commenced when the company Axis-Shield of Norway, and the University of Aarhus in Denmark, participated in a joint venture: Project E! 2263—HOLOTC “Development Of Diagnostic Test For Holotc”, from April 2000 to February 2005 (Eureka 2000). The stated objective of the project was to develop a new test that is simple and reliable for the diagnosis of vitamin B_12_ deficiency. Nexo et al. (2002b), from the University of Aarhus, reported a new method for measurement of HoloTC. Ulleland et al. (2002), sponsored by Axis-Shield, reported having developed the first reliable assay for HoloTC.

The HoloTC test as an alternative to the total vitamin B_12_ assay was further supported, from October 2002 to March 2006, by the European Union demonstration project: QLK3-CT-2002-01775 “Demonstration of the clinical utility of holotc as an early marker of vitamin b_12_ deficiency” (European Union 2002). The project was coordinated by the University of Aarhus in Denmark; other participants were Axis-Shield and the University of Bergen in Norway, the University of Oxford in the UK and Trinity College Dublin in Ireland. The stated aims of the project were to: “demonstrate the analytical performance of this assay in 4 countries; to show its superiority to conventional methods for assessment of vitamin B_12_ status; and to demonstrate its value in older patients with depression or dementia”.

As the commercial partner in the development of the HoloTC immunoassay, Axis-Shield has published several documents in support of the HoloTC test as an alternative to the total vitamin B_12_ assay (Axis-Shield 2007a, b, 2012, 2014a, b). In their presentation of February 2007, Axis-Shield cites Herbert’s model for the sequential development of vitamin B_12_ deficiency. A modified version of Herbert’s (1994) hypothesis, as a simplified diagram, was provided to support the claim that HoloTC is a more sensitive indicator of vitamin B_12_ deficiency than is total vitamin B_12_. The same diagram was used by Ralph Green in his detailed presentation to the *Specialty Labs Web Conference* of December 2007, when supporting the case for using HoloTC for the diagnosis of vitamin B_12_ deficiency (Green 2007).

Based on Herbert’s models, in which the HoloTC concentration falls as the vitamin B_12_ status progresses from *Normal* to *Negative Vitamin B*_12_*Balance* as the first evidence of deficiency, Axis-Shield (2007a) claims that “Active-B12 levels react early in the process” and “Active-B12 levels are low in patients with biochemical signs of vitamin B_12_ deficiency”.

Axis-Shield (2007a) cites Herrmann et al. (2003a), saying “Data suggests improved identification of B_12_ deficient patients with Active-B12 compared to total serum B_12_”.

Axis-Shield initially marketed their own immunoassay kit for HoloTC, then formed partnerships with manufacturers of automated immunoassay analysers. In 2006 Axis-Shield marketed their RIA (radioimmunoassay) kit for HoloTC (Axis-Shield 2007b), the first commercially available test for holotranscobalamin. In 2007 Abbott Laboratories, in partnership with Axis-Shield, commenced marketing their MEIA (Microparticle Enzyme Immunoassay) for assay of HoloTC on the AxSYM analyser (Abbott Laboratories 2006; Axis-Shield and Abbott Laboratories 2006a, b, 2007a, b; Brady et al. 2007, 2008). Pry (2006), for Abbott Laboratories, presented *Holo Transcobalamin; A Predictor of Vitamin B*_12_*Status*. Brady et al. (2008), employees of Axis-Shield, stated that “The AxSYM Active B12 assay allows rapid, precise, sensitive, specific, and automated measurement of human holoTC in serum and plasma”. In 2011 Abbott Laboratories broadened the application for the HoloTC kit to include their automated Architect high-throughput immunoassay analyser (Abbott Laboratories 2011). IBL International (2013) markets an enzyme-immunoassay (ELISA) kit, and Siemens is currently developing a HoloTC kit for use on their ADVIA Centaur immunoassay system.

## Review

### Summary of original research and previous reviews

Results from previous original research, for HoloTC sensitivity and specificity compared to total vitamin B_12_, are summarised in Table 2. Many authors concluded that HoloTC is an earlier and more sensitive indicator of the onset of vitamin B_12_ deficiency than total vitamin B_12_ (Table 3, column 1). Other original studies have concluded that there is no significant improvement in sensitivity of HoloTC in comparison to total vitamin B_12_ (Table 3, column 2). Quotations from original reports are available in tables in Microsoft Excel spreadsheet file, Additional file 3.

**Table 2:** Sensitivity and specificity—HoloTC versus B_12_—original research data

Reference	Sensitivity	Specificity	Cut-off	AUC
Bamonti et al. (2010)	0.74	0.52	HoloTC < 40 pmol/L	0.75
Clarke et al. (2007) (definite deficiency)	0.771 versus 0.757	0.761 versus 0.724	MMA > 0.75 μmol/L	0.85 versus 0.76
Clarke et al. (2007) (probable deficiency)	0.647 versus 0.626	0.792 versus 0.748	MMA > 0.45 μmol/L	0.79 versus 0.87
Goringe et al. (2006)			HoloTC < 38 pmol/L	0.75 versus 0.72
Heil et al. (2012)	0.83 versus 0.64	0.60 versus 0.64	MMA > 0.45 μmol/L	0.78 versus 0.70
Herrmann et al. (2003a)	0.87 versus 0.45	0.75 versus 0.98	MMA > 0.271 μmol/L	0.879 versus 0.836
Herrmann and Obeid (2013)	0.72 versus 0.72	0.54 versus 0.41	MMA > 0.300 μmol/L	0.714 versus 0.632
Hvas and Nexo (2003)	1.0	0.89		
Hvas and Nexo (2005)			MMA > 0.75 μmol/L	0.90 versus 0.85
Lindemans et al. (2007)			MMA > 0.26 μmol/L	0.80 versus 0.68
Lloyd-Wright et al. (2003)			MMA > 0.75 μmol/L	0.87 versus 0.86
Miller et al. (2006)			holoTC < 35 pmol/L	0.828 versus 0.816
Obeid and Herrmann (2007a)	0.72		MMA > 0.300 μmol/L	0.71 versus 0.60
Palacios et al. (2013)	0.44 versus 0.20	0.94 versus 0.94	HoloTC < 35 pmol/L	0.75 versus 0.69
Schrempf et al. (2011)	0.563 versus 0.662	0.505 versus 0.621	MMA > 47 μg/L	0.66 versus 0.72
Scott et al. (2007)			MMA > 0.75 μmol/L	0.85 versus 0.75, 0.74, 0.72
Valente et al. (2011)	0.55 versus 0.33	0.96 versus 0.95	Red cell cobalamin <33 pmol/L	0.90 versus 0.80

Clarke et al. (2007) compared the HoloTC immunoassay to two assay methods for total vitamin B_12_: Beckman and Centaur

Scott et al. (2007) compared the HoloTC immunoassay to three assay methods for total vitamin B_12_: Beckman, Centaur and micro assays

*AUC*, area under ROC curve for HoloTC or total vitamin B_12_; ROC curve, Receiver Operating Characteristic = sensitivity versus (1 − specificity) = true positive versus false positive (Miller et al. 2006); Cut-off, analyte and cut-off value used to define vitamin B_12_ deficiency

**Table 3:** Sensitivity and specificity—HoloTC versus B_12_—original research conclusions

Positive conclusions	Negative or neutral conclusions
Augoustides-Savvopoulou et al. (2007)	Al Aisari et al. (2010)
Bhat et al. (2009)	Chen et al. (2005)
Black et al. (2006)	Clarke et al. (2007)
Bor et al. (2004)	Goringe et al. (2006)
Čabarkapa et al. (2007)	Loikas et al. (2003)
Fragasso et al. (2012)	Loikas (2007)
Heil et al. (2012)	Miller et al. (2006)
Herrmann et al. (2003a)	Nilsson et al. (2004)
Herrmann et al. (2005)	Palacios et al. (2013)
Herrmann and Obeid (2013)	Remacha et al. (2014)
Hvas and Nexo (2003)	Schrempf et al. (2011)
Hvas and Nexo (2005)	Sobczyńska-Malefora et al. (2014)
Lee et al. (2009)	van Asselt et al. (2003)
Lindemans et al. (2007)	
Lindgren et al. (1999)	
Lloyd-Wright et al. (2003)	
Lobreglio et al. (2008)	
Morkbak et al. (2007)	
Nexo et al. (2002a)	
Obeid and Herrmann (2007a)	
Scott et al. (2007)	
Serefhanoglu et al. (2008)	
Sikaris (2010)	
Valente et al. (2011)	
Vanpoucke et al. (2007)	
Woo et al. (2010)	

Positive, neutral or negative conclusions are as understood by this author to be the intention of authors of cited article

Quotations from original reports are available in tables in Microsoft Excel spreadsheet file, Additional file 3

In March 2012, a group of nine internationally recognised experts issued an *Expert’s Consensus Statement* in support of the use of the HoloTC test: “Emerging evidence indicates that holotranscobalamin (Active-B12) is a more reliable marker of a patient’s B_12_ status than is serum vitamin B_12_” (Herrmann et al. 2012) (Table 4). However, there is not consensus between all experts, as demonstrated by a report on the NHANES (National Health and Nutrition Survey) roundtable on biomarkers of vitamin B_12_ status (Yetley et al. 2011). The report recommended total vitamin B_12_ as the marker for circulating vitamin B_12_ because of the need for additional performance studies of the HoloTC test.

**Table 4:** Signatories to “Expert’s Consensus Statement”

Signatory	Institution
Wolfgang Herrmann	Saarland University
Rima Obeid	Saarland University
Ralph Green	University of California
Donald Jacobsen	Cleveland Clinic
Dominic Harrington	St. Thomas’ Hospital
Per Magne Ueland	University of Bergen
Jan Lindemans	Erasmus Medical Center
Ebba Nexø	Aarhus University Hospital
Anne Molloy	Trinity College Dublin

From Herrmann et al. (2012)

Consensus Statement: “Emerging evidence indicates that holotranscobalamin (Active-B12) is a more reliable marker of a patient’s B_12_ status than is serum vitamin B_12_”

Reviews, presentations, commentary and letters, on the use of HoloTC as an earlier and more sensitive indicator of vitamin B_12_ deficiency than total vitamin B_12_, have been mixed. Several authors supported the claim that HoloTC was superior (Green 2011; Greibe et al. 2012; Nexo and Hoffmann-Lücke 2011). Others raised concerns about the clinical utility of the test (Carmel 2002, 2011, 2012; Devalia 2006; Devalia et al. 2014; Hvas and Nexo 2006; Oberley and Yang (2013). Herrmann et al. (2003b) and Herrmann and Obeid (2007, 2008) recommended the combined use of methylmalonic acid (MMA) and HoloTC. Questioning the superiority of the HoloTC test, Carmel (2002) stated that “what low holo-TC concentrations really tell us remains elusive.”

### HoloTC problems

There are four problematic aspects of HoloTC, where there are major differences between authors: the value of the half-life of HoloTC; the correlation between HoloTC and total vitamin B_12_; sensitivity of HoloTC to recent absorption of vitamin B_12_; the value of HoloTC cut-off to detect negative vitamin B_12_ balance. The latter two differences are the most important because they raise the questions of what it is that HoloTC concentration actually indicates, and whether or not it is possible to use it to reliably detect the early onset of vitamin B_12_ deficiency.

#### Half-life

Although the proponents of HoloTC, as an early indicator of vitamin B_12_ deficiency, cite the shorter half-life of HoloTC compared to HoloHC as a factor, there is no general agreement on the absolute value (Table 5). In supporting their case for HoloTC, Axis-Shield (2007b) states: “The markedly shorter half-life for HoloTC of 1–2 hours versus 9 days for HoloHC makes a decrease of HoloTC one of the earliest markers of cobalamin deficiency”; Herbert (1994) gives values of 6 min for HoloTC and 240 h for HoloHC. Whether the half-life of HoloTC is minutes or hours is not significant compared to the generally agreed 240 h for HoloHC. Therefore, although the differences between authors are considerable and should be resolved, they do not invalidate the case for using HoloTC.

**Table 5:** Half-life of HoloTC

Reference	Half-life HoloTC
Axis-Shield (2007b)	1–2 h
Bor et al. (2004)	1–12 h
Carmel and Agrawal (2012)	Minutes
Chatthanawaree (2011)	1 h
Chen et al. (2005)	Few hours
Green (2007)	6 min
Herbert (1994)	6 min
Herrmann et al. (2003a)	6 min
Hvas et al. (2005)	1–2 h
Lindgren et al. (1999)	1–2 h
Loikas et al. (2003)	60 min
Quadros (2010)	1–2 h
von Castel-Roberts et al. (2007)	18 h

#### Correlation with total vitamin B_12_

The correlation between HoloTC and total vitamin B_12_ concentrations, in reports of original research, varies widely between authors (Table 6). Bamonti et al. (2010) reported a weak correlation (r = 0.42) whereas Augoustides-Savvopoulou et al. (2007) reported a very strong correlation (r = 0.882). Although most researchers reported a single value for r, there were notable exceptions. Herrmann and Obeid (2013) reported separate values of r for low or high values of total vitamin B_12_ (r = 0.524 for low B_12_; r = 0.403 for high B_12_). Refsum et al. (2006) reported separate values for male and female subjects (r = 0.65 for male; r = 0.61 for female). The differences between findings, for correlation coefficient, are important because they reflect different sensitivity and/or specificity relationships between HoloTC and total vitamin B_12_. A very high correlation, over a wide range of values, would imply that HoloTC could not detect the onset of vitamin B_12_ deficiency any earlier than total vitamin B_12_. However, a very low correlation would not necessarily mean that HoloTC is a more sensitive marker of the transition from normal to negative vitamin B_12_ balance.

**Table 6:** Correlation between total vitamin B_12_ and HoloTC

Reference	HoloTC versus B_12_ correlation coefficient (r)
Al Aisari et al. (2010)	0.765
Augoustides-Savvopoulou et al. (2007)	0.882
Bamonti et al. (2010)	0.42
Čabarkapa et al. (2007)	0.53
Chen et al. (2005)	0.45
Clarke et al. (2007)	0.61
Fragasso et al. (2012)	0.64
Goringe et al. (2006)	0.63 (r^2^ = 0.397)
Herrmann et al. (2003a)	0.75
Herrmann and Obeid (2013)	0.577 (whole), 0.524 (low B_12_), 0.403 (high B_12_)
Hvas and Nexo (2005)	0.71
Lee et al. (2009)	0.6591
Lloyd-Wright et al. (2003)	0.75
Lobreglio et al. (2008)	0.495
Loikas et al. (2003)	0.80
Loikas (2007)	0.78
Nexo et al. (2002b)	0.45
Palacios et al. (2013)	0.65
Refsum et al. (2006)	0.65 (male), 0.61 (female)
Schrempf et al. (2011)	0.577–0.637
Scott et al. (2007)	0.5
Vanpoucke et al. (2007)	0.53 (r^2^ = 0.28)

Herrmann and Obeid (2013) reported three values for r: for the whole range of total vitamin B_12_; for low values of vitamin B_12_; for high values of vitamin B_12_

Refsum et al. (2006) reported separate values for r for males and females

#### Sensitivity to recent absorption

The reported sensitivity of the HoloTC concentration to recent absorption of vitamin B_12_ varies between authors (Table 7), leading to the important question about whether HoloTC concentration indicates long-term status or recent absorption of vitamin B_12_ or both. Axis-Shield and Abbott Laboratories (Axis-Shield and Abbott Laboratories 2006a, 2007a; Axis-Shield 2007a) cite Chen et al. (2005), stating that HoloTC reflects vitamin B_12_ status “independent of recent absorption of the vitamin”. Chen et al. (2005) actually concluded that “Metabolic cobalamin status is a major determinant of serum holo-TC II. Absorption status may have mild influence as well”. Axis-Shield (2012) cite Nexo: “newly absorbed vitamin B_12_ occurs as holoTC, and therefore an increase in holoTC upon oral loading with vitamin B_12_ can be used to judge the capacity for uptake of the vitamin”. Several authors found the issue to be problematic (Bamonti et al. 2010; Loikas 2007; Carmel 2002, 2012). Bamonti et al. (2010) cited the conflicting findings of Chen et al. (2005) and Bor et al. (2004). Loikas (2007) commented on the apparently contradictory promotion of HoloTC as “the most sensitive and specific indicator of early vitamin B_12_ deficiency” and the proposed use of HoloTC as “a marker of vitamin B_12_ absorption”, concluding that “Reconciling these two phenomena is problematic because, although frequently coexisting, they are not identical”. In his editorial, Carmel (2002) observed that “The favoured hypotheses have been that low holo-TC is either an early sign of general cobalamin insufficiency or specific evidence of decreased absorption of cobalamin. The distinction between these two very separate explanations has blurred, particularly as advocacy became more enthusiastic, but it is not an idle distinction. This central issue and many other questions need resolution”.

**Table 7:** Sensitivity to recent absorption

Reference	Dependence on recent absorption
Axis-Shield and Abbott Laboratories (2006a)	Independent
Axis-Shield and Abbott Laboratories (2007a)	Independent
Axis-Shield (2007a)	Independent
Axis-Shield (2012)	Dependent
Bamonti et al. (2010)	Problematic
Bhat et al. (2009)	Dependent
Bor et al. (2004)	Dependent
Bor et al. (2005)	Dependent
Carmel (2002)	Problematic
Carmel (2012)	Problematic
Chen et al. (2005)	Both?
Green (2011)	Both
Hvas et al. (2005)	Independent
Hvas et al. (2007)	Dependent
IBL International (2013)	Independent
Lindgren et al. (1999)	Dependent
Loikas (2007)	Problematic
Morkbak et al. (2005)	Dependent
Nexo et al. (2002a)	Dependent
Nexo and Hoffmann-Lücke (2011)	Both
Robinson et al. (2011)	Both
von Castel-Roberts et al. (2007)	Dependent

Quotations from original reports are available in tables in Microsoft Excel spreadsheet file, Additional file 3

#### No agreed cut-off

Another important problem is that there is no agreement on a single value for the cut-off level for the minimum concentration of HoloTC, as required to distinguish between normal vitamin B_12_ status and the first stage of deficiency (Table 8). Morkbak et al. (2005) quoted a range of 11–41 pmol/L for eight European studies, stating: “There is no consensus concerning the choice of reference intervals for holoTC”. According to Heil et al. (2012): “large discrepancies exist with regard to the choice of cut-off value for HoloTC (range 20–45 pmol/L), which mak*es data* interpretation difficult”. In their review, Aparicio-Ugarriza et al. (2014) found that, in 69 studies between 1992 and 2014, researchers quoted HoloTC concentration cut-offs from 20 to 50 pmol/L.

**Table 8:** HoloTC reference intervals and cut-offs

Reference	Reference interval (pmol/L)	Cut-off (pmol/L)
Abbott Laboratories (2006)		37
Abbott Laboratories (2011)		35
Al Aisari et al. (2010)	9–123	
Aparicio-Ugarriza et al. (2014)	20–50 (literature review)	
Augoustides-Savvopoulou et al. (2007)		35
Axis-Shield and Abbott Laboratories (2006a)		35
Axis-Shield (2007a)		35
Axis-Shield (2007b)		37
Bamonti et al. (2010)		40
Black et al. (2006)	23–100	
Brady et al. (2007)		35
Chen et al. (2005)	46–356	30
Clarke et al. (2007)		45
Fragasso et al. (2012)		35
Goringe et al. (2006)	16 (diagnostic), 38 (laboratory)	
Green (2007, 2011)		35
Heil et al. (2012)		32
Herbert (1987)		22
Herbert (1994)		37
Herrmann et al. (2003a, b, 2005)		35
Herrmann and Obeid (2007)	40–70 “Grey zone”	
Herrmann and Obeid (2008)		35
Herrmann and Obeid (2013)	22–76 “Grey zone”	
Herrmann et al. (2003a)		35
Herrmann et al. (2005)		35
Hooshmand et al. (2012)		35
Hvas and Nexo (2003)		50
Hvas and Nexo (2005)		40
Lee et al. (2009)		42.48
Lindemans et al. (2007)	20–122	
Lindgren et al. (1999)	35–160	
Lloyd-Wright et al. (2003)	<25 likely, >50 unlikely B_12_ deficiency	
Lobreglio et al. (2008)		35
Loikas et al. (2003, 2007a, b)		37
Loikas (2007)	37–171	
Miller et al. (2006)		35
Morkbak et al. (2005)	40, 11–41 (literature review)	37
Morkbak et al. (2007)	40–150	
Nexo et al. (2002a)	40–150	
Nexo et al. (2002b)	40–150	
Nexo and Hoffmann-Lücke (2011)	40–200	
Obeid and Herrmann (2007a, 2007b)		35
Palacios et al. (2013)		35
Pry (2006)		37
Refsum et al. (2006)	42–157	
Remacha et al. (2014)		33.5
Schrempf et al. (2011)		42
Serefhanoglu et al. (2008)		37
Sobczyńska-Malefora et al. (2014)	25–50 “poor predictor MMA”	
Valente et al. (2011)	20–30 “indeterminate zone”	20
van Asselt et al. (2003)	38–113	
Vanpoucke et al. (2007)		37
Woo et al. (2010)		35

Furthermore, several researchers quote a *grey zone*, or indeterminate range of cut-off values for HoloTC concentration (Table 8). Herrmann and Obeid (2007) quote a *grey zone* for HoloTC cut-offs, of 40–70 pmol/L; they later changed this to 23–75 pmol/L (Herrmann and Obeid 2013). Lloyd-Wright et al. (2003) say that deficiency is likely for a HoloTC concentration <25 pmol/L, and unlikely for >50 pmol/L, leaving an indeterminate range of 25–50 pmol/L. Sobczyńska-Malefora et al. (2014) found that HoloTC concentrations in the range of 25–50 pmol/L could not be used to predict the vitamin B_12_ status as defined by MMA concentration. Valente et al. (2011) reported an *indeterminate zone* of 20–30 pmol/L for HoloTC concentrations. In their BJH guideline, Devalia et al. (2014) recommended that “Serum holotranscobalamin has the potential as a first-line test, but an indeterminate ‘grey area’ may still exist”.

The HoloTC test appears to suffer from a similar limitation to that found for total vitamin B_12_; the range of normal values for an individual is much narrower than the range of normal for a population (Herbert 1987). According to McCaddon et al. (2003), referring to the HoloTC test: “the dispersion of values for any individual will span only a small part of any reference interval”.

The absence of an agreement on a single cut-off value for HoloTC, for diagnosis of vitamin B_12_ deficiency, is inconsistent with a widely stated reason for supporting the use of this test in place of total vitamin B_12_: that there is a wide indeterminate range for total vitamin B_12_. In promoting their HoloTC assay, Axis-Shield (2007a) state this inadequacy of the total vitamin B_12_ test: “There is a grey zone between approximately 151–300 pmol/L B_12_ where there is likely to be misclassification of B_12_ status if relying on total serum B_12_ alone.” If there is no universally agreed single cut-off value for a minimum HoloTC concentration, this test appears to have the same flaw as total vitamin B_12_. Is it possible to reliably detect the early onset of vitamin B_12_ deficiency, i.e. the transition between Herbert’s sequential stages from *Normal* to *Early Negative B*_12_*Balance*, if there is no agreed single cut-off value for HoloTC?

### Potential for data manipulation to promote HoloTC

#### Area Under Curve (AUC)

Where the Area Under Curve (AUC) is reported for Receiver Operating Characteristic (ROC) curves, the selection of the reference analyte and value of the reference cut-off determines the relative areas under the curve (Table 2). For example, Heil et al. (2012) produced three ROC curves where different cut-off levels of MMA were used to define vitamin B_12_ deficiency, demonstrating how the choice of reference cut-off level determines the difference in AUC for HoloTC compared to total vitamin B_12_. With the lowest MMA cut-off, of >0.32 µmol/L, the AUC was 0.70 for HoloTC, compared to 0.63 for total vitamin B_12_. At their preferred MMA cut-off, of >0.45 µmol/L, the AUC was 0.78 for HoloTC, compared to 0.70 for total vitamin B_12_. When the MMA cut-off was increased to >0.77 µmol/L, the AUC was 0.92 for HoloTC, compared to 0.73 for total vitamin B_12_.

By increasing the MMA cut-off, the difference in AUC between HoloTC and total vitamin B_12_ is increased (Heil et al. 2012, Figure 1), improving the apparent performance of HoloTC, but many more vitamin B_12_ deficient patients will be misdiagnosed as normal. The next stage of the process, selection of the total vitamin B_12_ and HoloTC cut-offs, is a trade-off between sensitivity and specificity (Heil et al. 2012, Figure 4).

#### The trade-off between sensitivity and specificity

As noted by this author (Golding 2016), it is possible to alter the apparent relative sensitivity of any pair of analytes by selectively changing the cut-off value of one or both of them. As demonstrated in that experiment, selecting different cut-off values for total vitamin B_12_ changed the relative sensitivities of HoloTC and total vitamin B_12_; the same applies to the cut-off value for HoloTC. When the HoloTC cut-off is increased, the sensitivity to vitamin B_12_ deficiency is increased, but the specificity of the test is reduced, increasing the number of false positive results. Conversely, if the HoloTC cut-off is decreased, the specificity of the test is increased, but the sensitivity of the test is reduced, increasing the number of false negative results.

From their ROC charts, Heil et al. (2012, Figure 3) selected MMA > 0.45 µmol/L to define vitamin B_12_ deficiency. With a HoloTC cut-off value of <21 pmol/L, the specificity was impressively high at 88 % but the sensitivity was only 64 % (Heil et al. 2012, Figure 4b). By selecting the best trade-off between sensitivity and specificity for HoloTC, with a HoloTC cut-off value of <32 pmol/L, they obtained an impressive HoloTC sensitivity of 83 % but a specificity of only 60 %. To overcome this problem, as did Herrmann and Obeid (2007, 2008), the authors suggest using MMA as a secondary test to confirm vitamin B_12_ deficiency in cases where HoloTC is <32 pmol/L. This approach would have failed to detect any vitamin B_12_ deficiency in the subject of this author’s experiment (Golding 2016) because, despite overt metabolic disturbance evident from raised MMA concentration, and the onset of severe symptoms including peripheral neuropathy, HoloTC concentration never fell below 33 pmol/L.

### Contrary findings

The results of several studies did not support the claims that HoloTC is a significantly earlier marker of vitamin B_12_ deficiency than total B_12_ (Clarke et al. 2007; Palacios et al. 2013; Schrempf et al. 2011; Miller et al. 2006; Remacha et al. 2014). This author, in his single-subject self-experiment, reported no significant difference in response between HoloTC and total vitamin B_12_ (Golding 2016).

Contrary to Heil et al., Schrempf et al. (2011) reported a lower AUC for HoloTC than for total vitamin B_12_; 0.563 versus 0.662 (Table 2). Their study was conducted on neuropsychiatric patients with vitamin B_12_ deficiency defined by MMA > 47 µg/L (40 µmol/L). The authors presented the ROC curves for three patient groups: all patients; those with classic vitamin B_12_ deficiency (subacute combined degeneration and/or peripheral neuropathy); those with peripheral neuropathy only. In each grouping, the AUC for total vitamin B_12_ was greater than for HoloTC (Schrempf et al. 2011, Figure 1).

Based on their ROC results, in which the AUCs for HoloTC and total vitamin B_12_ were 0.828 and 0.816 respectively, Miller et al. (2006) concluded that “HoloTC and total vitamin B12 have equal diagnostic accuracy in screening for metabolic vitamin B_12_ deficiency”. They also reported correlations between their results, for HoloTC and total vitamin B_12_, which were not consistent with Herbert’s model. For example, some patients with normal HoloTC (>35 pmol/L) were low in total vitamin B_12_ (<148 pmol/L).

As observed by Herrmann and Obeid (2013), in reporting results of their investigation of the biological markers of vitamin B_12_ deficiency, “The shape of the ROC curve illustrates that the holoTC test is not sufficient to separate between deficient and non-deficient individuals with high reliability”.

Remacha et al. (2014) investigated 106 patients with total vitamin B_12_ concentration ≤200 pmol/L, defining low HoloTC as a concentration <33.5 pmol/L and high MMA as a concentration >0.40 µmol/L. Of the 31 patients with normal HoloTC concentrations, 13 had high levels of MMA; of the 75 patients with low HoloTC concentrations, 27 had normal MMA. Remacha et al. reported that “HoloTC was not decreased in one-third of patients with low Cbl, but MMA/Hcy levels were elevated in half of them, reflecting Cbl deficiency.”

### The problems with Herbert’s model

The absence of an agreed single value for the HoloTC cut-off, together with uncertainty about whether HoloTC represents recent absorption of vitamin B_12_ or long-term status or both, must raise questions about Herbert’s hypothesis. Some of the most recent original research challenges Herbert’s model for the “Sequential stages in the development of vitamin B_12_ deficiency”. In reporting their original research results, Remacha et al. (2014) commented that “These data do not support holoTC as the earliest marker of Cbl deficiency and challenge the classification in stages of Cbl deficiency”.

In Herbert’s revised 1994 model (Herbert 1994), the change from *Normal* to Early *Negative Vitamin B*_12_*Balance* is marked by a fall in HoloTC concentration from >37 to <30 pmol/L. Even if it is assumed that such a highly precise and accurate routine clinical assay is possible, there remain two major unresolved fundamental problems with Herbert’s hypothesis.

Firstly, as raised by several authors, there is uncertainty about what HoloTC actually represents (Table 7). Does HoloTC concentration indicate recent absorption of vitamin B_12_, long-term body store or both? As stated by Chen et al. (2005): “The concept that a test can be used to diagnose both deficiency and malabsorption is problematic because the 2 defects are not identical. If holo-TC II truly reflects both processes, holo-TC II changes would perforce lose all diagnostic specificity for either process”. For the HoloTC immunoassay to have any clinical value in detecting the earliest onset of vitamin B_12_ deficiency, as Herbert intended, it would need to be either sensitive only to long-term status or be performed after a well-defined period of fasting.

Secondly, the model demands the reliable detection of a change in HoloTC concentration far smaller than the reported range of cut-off values (Table 8). As noted by Sobczyńska-Malefora et al. (2014), “there has been little consensus with regard to the assigned cut-off to discriminate between replete and deficient states”. Herbert’s required change in HoloTC concentration from >37 to <30 pmol/L, to detect the earliest onset of vitamin B_12_ deficiency, is inconsistent with the reported range of cut-off values from 20 to 50 pmol/L. Furthermore, the use of various *grey zones*, for HoloTC concentration (Table 8), is inconsistent with Herbert’s hypothesis in which *Early Negative Vitamin B*_12_*Balance* is detected by a universally agreed, and closely specified, fall in HoloTC concentration.

### Why Herbert’s model is flawed

Herbert’s model for the staged development of vitamin B_12_ deficiency is based on his erroneous hypothesis that HoloTC will always be the first analyte to respond to a deficiency, and that a minimum normal concentration of HoloTC may be universally defined. How Herbert developed his hypothesis, and why it is flawed, is best explained by examining the process of gastrointestinal absorption and transport of vitamin B_12_ in humans.

#### Normal gastrointestinal absorption of vitamin B_12_

**Fig. 3:**
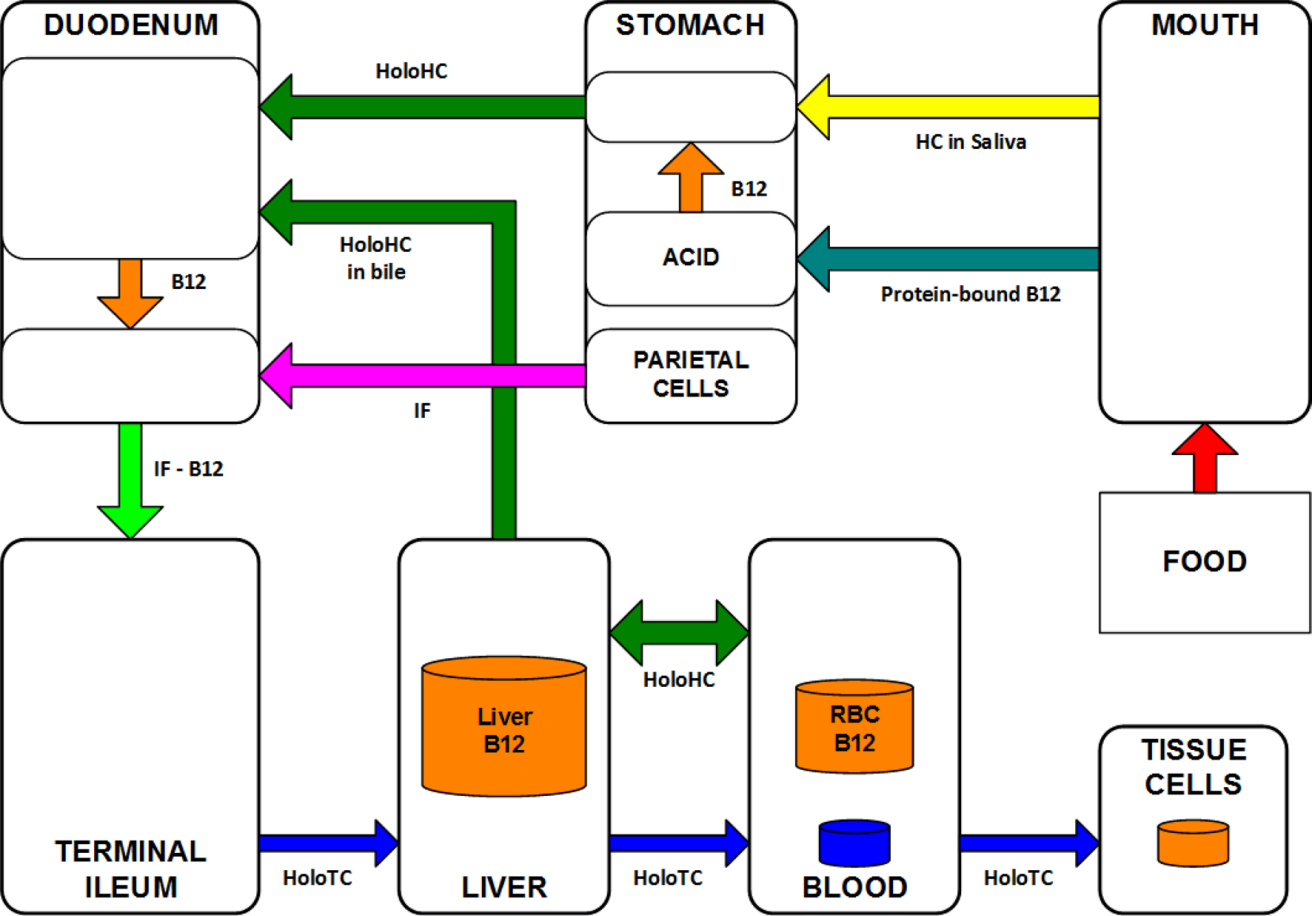
Vitamin B_12_ gastrointestinal absorption. Derived from Seetharam and Yammani (2003, Figure 1), Andrès et al. (2004, Figure 1) and Loikas (2007, Figure 2.4)

Figure 3 is a simplified diagram containing the main elements of the process of gastrointestinal absorption and transport of vitamin B_12_ in humans. In a healthy person, stomach acid separates food vitamin B_12_ from protein; haptocorrin (HC) from saliva then binds with the free vitamin B_12_ to form the holohaptocorrin (HoloHC) (Loikas 2007, Figure 2.4). The increased pH in the duodenum causes the vitamin B_12_ to be released from the HC; intrinsic factor (IF), produced by parietal cells in the stomach, combines with the vitamin B_12_ in the duodenum to produce IF-B12 (Loikas 2007, Figure 2.4). The IF is then degraded in the terminal ileum, producing free vitamin B_12_ (Seetharam and Yammani 2003, Figure 1; Andrès et al. 2004, Figure 1; Loikas 2007, Figure 2.4).

There is no consensus on the exact location of the production of HoloTC and HoloHC.

According to Seetharam and Yammani (2003, Figure 1), and Andrès et al. (2004, Figure 1), the free B_12_ is bound to TC in the ileum to form HoloTC; they show HoloHC in the portal vein but do not say how or where it is formed. Quadros (2010) states that “The IF-Cbl absorbed in the distal ileum appears in the circulation bound to TC”, implying that all of the free B_12_ is bound to TC to form HoloTC; it is unclear from this whether the free B_12_ binds to the TC in the ileum or in the circulation. According to Furger (2012) “In the blood plasma Cbl is bound either to HC or TC, where only TC is responsible for the Cbl uptake into cells”; again, it is unclear where the binding takes place. If all of the free vitamin B_12_ in the ileum is bound to TC, regardless of whether the binding takes place in the ileum or in the circulation, then the HoloHC in the circulation must ultimately be produced from vitamin B_12_ freed from the HoloTC.

The HoloTC enters the general circulation from where it is delivered to cells for metabolism (Seetharam and Yammani 2003, Figure 1; Andrès et al. 2004, Figure 1; Loikas 2007, Figure 2.4; Quadros 2010; Furger 2012).

The HoloHC is passed, via the portal vein, to the liver where the vitamin B_12_ is separated from the HC and stored in the liver (Seetharam and Yammani 2003, Figure 1). When HoloTC is required for metabolism, some of the vitamin B_12_ stored in the liver is bound to HC and recycled via the bile (Seetharam and Yammani 2003, Figure 1; Loikas 2007, Figure 2.4).

#### Normal enterohepatic recycling of vitamin B_12_

Of crucial importance to this entire gastrointestinal absorption process is the enterohepatic recycling of the vitamin B_12_, shown in Fig. 3 as the transfer of HoloHC from the liver to the duodenum, in the bile. This recycling is not only essential for the efficient maintenance of the body store of vitamin B_12_; it is the means by which the serum concentration of HoloTC is regulated. The rate of transfer to the duodenum of HoloHC in bile, and the IF from the parietal cells, controls the rate of HoloTC production.

**Fig. 4:**
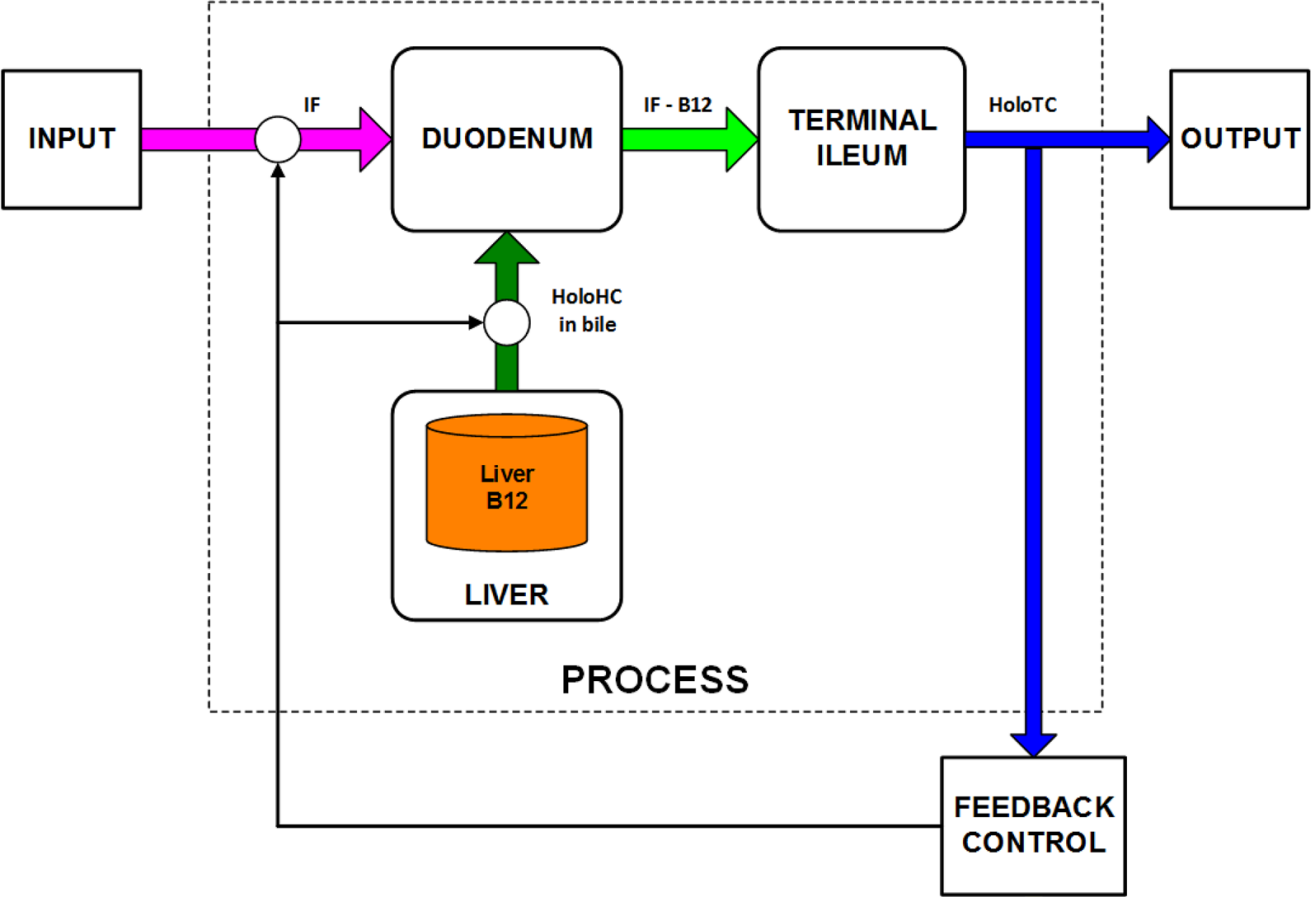
HoloTC feedback control. Derived from Seetharam and Yammani (2003, Figure 1), Andrès et al. (2004, Figure 1) and Loikas (2007, Figure 2.4)

This enterohepatic recycling process is shown, as a feedback control system, in Fig. 4. Here, the *Input* to the system is the intrinsic factor; the system *Output* is the HoloTC. The *Process* is the production of IF-B12 from the IF and vitamin B_12_ in the duodenum, and the binding of the B_12_ freed from IF in the terminal ileum with transcobalamin (TC) to produce holotranscobalamin (HoloTC). For the purposes of this discussion, the liver vitamin B_12_ store is not part of the input but considered to be a constant in the short-term. The *Feedback* signals control the rate of the process; details of the nature of these signals are beyond the scope of this review.

#### Failure of enterohepatic recycling and HoloTC production

If any one of the elements of this feedback control system suddenly fails entirely, the enterohepatic recycling will stop immediately, and no HoloTC will be produced; for example, if the supply of intrinsic factor or production of bile suddenly stops or if the terminal ileum or duodenum are removed. Under these circumstances, Herbert’s hypothesis would be expected to hold true in one respect; the fall in HoloTC would be an early indicator of a vitamin B_12_ deficiency. The HoloHC has a far longer half-life than the HoloTC in serum, and there is a large store of vitamin B_12_ in the liver and red-cells, so HoloHC would fall much more slowly than HoloTC.

It is important to note that only a disturbance of the IF input, or of elements within this HoloTC feedback system, will affect its short-term performance. Assuming that the liver vitamin B_12_ store is full, any failure ahead the input of IF, or after the output of HoloTC will have no short-term effect on the production of HoloTC. This means that HoloTC will not be an early responder for all causes of vitamin B_12_ deficiency; if the enterohepatic cycle is not disrupted, HoloTC would remain normal until the liver store of HoloHC is exhausted.

#### HoloTC response depends on cause of vitamin B_12_ deficiency

The following discussion is based on this author’s interpretation of available information about the operation of the vitamin B_12_ enterohepatic cycle, and the response of total vitamin B_12_, HoloTC, tHcy and MMA for different causes of vitamin B_12_ deficiency. Human experimentation is needed to investigate the actual responses of the analytes under these various conditions.

According to Herbert (1987, 1994), there are six possible causes of vitamin B_12_ deficiency; three inadequacies and three excesses. These conditions, and their effect on the HoloTC feedback system, are listed in Table 9. A seventh very common cause of vitamin B_12_ deficiency, food-cobalamin malabsorption, was reported by Carmel (1995) and reviewed by Andrès et al. (2004); this is shown in Table 9 as a fourth inadequacy.

**Table 9:** Causes of vitamin B12 deficiency, and short-term effect on HoloTC production

Cause of vitamin B12 deficiency	Classification	Short-term effect on HoloTC production?
Inadequate dietary intake	Inadequacy	No
Defective gastrointestinal absorption (other than food-cobalamin absorption)	Inadequacy	Yes?
Defective gastrointestinal absorption (food-cobalamin malabsorption)	Inadequacy	No
Inadequate cellular utilization (deficiency of a cobalamin coenzyme)	Inadequacy	No
Increased requirement (pregnancy or hyperthyroidism)	Excess	No
Increased excretion (alcoholism)	Excess	No
Increased destruction (nutrient or drug interactions)	Excess	No, unless intrinsic factor destroyed

From Herbert (1987, 1994), Carmel (1995) and Andrès et al. (2004)

A suddenly inadequate dietary intake, for example commencing a vegan diet, will not affect the enterohepatic cycle because it does not affect the input of intrinsic factor into the cycle until the store of HoloHC is exhausted i.e. it occurs ahead of the cycle. Similarly, the onset of inadequate utilization in the cells also will not affect the production of HoloTC i.e. it occurs after the cycle. In either case, Herbert’s model will not apply because total vitamin B_12_ will fall before HoloTC responds, and MMA and tHcy will commence to increase immediately after HoloTC falls below normal for the individual.

An increased requirement, increased excretion or increased destruction, would tend to cause an increase in the rate of enterohepatic recycling to maintain the serum concentration of HoloTC; if all else is equal, the cycle will continue to produce HoloTC until the supply of stored HoloHC is consumed. Again, Herbert’s model will not hold true because total vitamin B_12_ will fall before HoloTC responds, and MMA and tHcy will commence to increase immediately after HoloTC falls below normal for the individual.

One inadequacy, gastrointestinal malabsorption, affecting the supply of intrinsic factor, production of bile or absorption of the IF-B12, has the potential to halt HoloTC production. HoloTC is likely to be a fast responder to a rapid failure such as the removal of the relevant part of the stomach, containing the parietal cells, or the removal of the duodenum or terminal ileum. In this case, however, MMA and tHcy would be expected to almost immediately increase above normal for the individual, so there would be no period when HoloTC is depleted without any metabolic disturbance, and Herbert’s model would not hold true.

If there is a gradual failure of supply of intrinsic factor to the input, in the case of Pernicious Anaemia where the autoimmune disease causes antibodies to attack the parietal cells or intrinsic factor or other slowly developing problem with gastrointestinal vitamin B_12_ absorption, the metabolic disturbance would again commence immediately but MMA and tHcy would increase more slowly. Again, because there would be no period when HoloTC is depleted without any metabolic disturbance, Herbert’s model would not apply.

The very common case of food-cobalamin malabsorption, where gastric atrophy occurs during ageing, was first identified after Herbert proposed his model (Carmel 1995; Andrès et al. 2004). This case would be similar to that of inadequate dietary intake, where the enterohepatic cycle is not disturbed because there is no effect on the input of intrinsic factor into the cycle until the liver store of HoloHC is exhausted. Because the deficiency occurs ahead of the cycle, there would be no period when HoloTC is depleted without any metabolic disturbance, so Herbert’s model would not hold true. If the slow onset of vitamin B_12_ deficiency in this case does cause HoloTC to fall significantly, and remain depleted before other analytes react, the published experimental results should demonstrate this. According to Andrès et al. (2004), >60 % of cases of vitamin B_12_ deficiency in the elderly are caused by food-cobalamin malabsorption. The studies reported by Miller et al. (2006), Clarke et al. (2007), Schrempf et al. (2011), Palacios et al. (2013) and Remacha et al. (2014) all involved aged patients, so the majority were therefore likely to have their vitamin B_12_ deficiency caused by food-cobalamin malabsorption. If Herbert’s model applied to those patients, HoloTC should have been reported as significantly more sensitive than total vitamin B_12_, but this was not the case in those studies.

## An alternative hypothesis

### Introduction

**Fig. 5:**
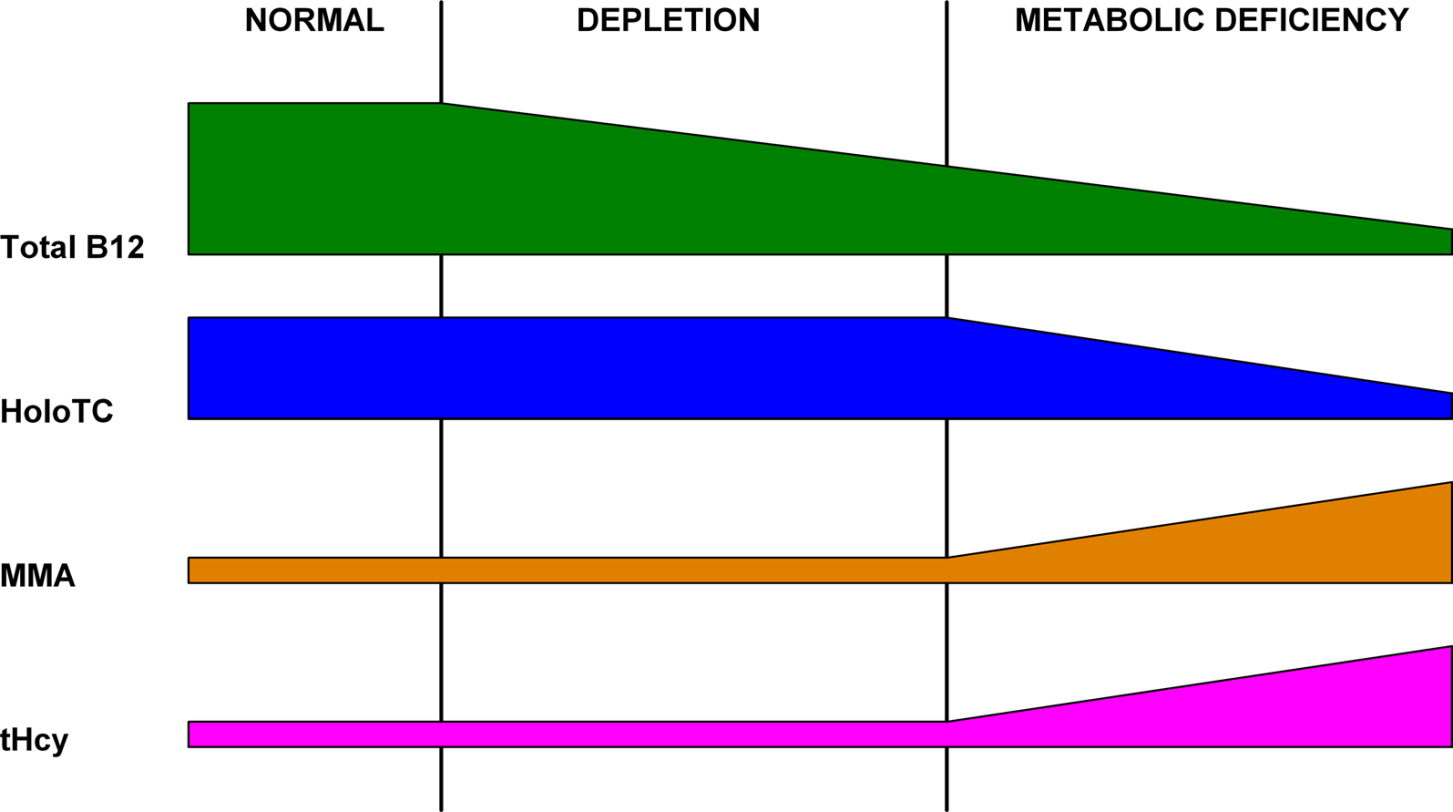
Alternative model for the development of vitamin B_12_ deficiency. Total B_12_, serum total vitamin B_12_ concentration; HoloTC, serum holotranscobalamin concentration; MMA, plasma methylmalonic acid concentration; tHcy, plasma total homocysteine concentration

This author proposes an alternative model (Fig. 5) in which the earliest onset of vitamin B_12_ deficiency, or the transition from *Normal* to *Early Negative Vitamin B*_12_*Balance*, is not defined by a fall in HoloTC concentration from above one universally specified level to below another. Instead, vitamin B_12_ deficiency is defined by a change, in total vitamin B_12_, HoloTC or metabolite concentrations, from what is normal for the individual.

### Stages of development of vitamin B_12_ deficiency

The alternative model for the development of vitamin B_12_ deficiency contains only three stages: *Normal*; *Depletion*; *Metabolic Deficiency* (Fig. 5). The *Normal* stage is defined as the condition in which the intake, and the internal processing, for an individual provides sufficient vitamin B_12_ to sustain all of their metabolic requirements and maintain their body store of vitamin B_12_. The *Depletion* stage, marked by the commencement of a long-term fall in serum total vitamin B_12_ concentration, is defined as the condition in which the intake, or the internal processing, for an individual does not provide sufficient vitamin B_12_ to sustain all of their metabolic requirements, which are then maintained by depleting their body store of vitamin B_12_. The *Metabolic Deficiency* stage, marked by the commencement of a fall in the HoloTC concentration and an increase in either MMA or tHcy or both metabolites, is defined as the condition in which the depleted body store of vitamin B_12_, or the internal processing, for an individual is insufficient to sustain all of their metabolic requirements.

### Cellular storage of vitamin B_12_ and potential delays in metabolic response to fall in HoloTC

Herbert (1994) stated: “holoTCII will always be low before there is a rise in methylmalonate or homocysteine if that rise is due to a vitamin B-12 deficiency.” This alternative model proposes an additional principle; that any fall in HoloTC, to below normal for the individual, must result in a disturbed metabolism and a rise in the metabolites. How quickly this happens depends on how much vitamin B_12_ is stored in the cells; this varies between cell types; some cells, including nerve cells and some blood cells, have very small vitamin B_12_ stores. For example, as stated by Herbert (1994) “Lack of delivery of vitamin B-12 to the glial cells of the brain quickly wipes out their small vitamin B-12 stores, after which they become vitamin B-12 deficient”.

This alternative hypothesis therefore assumes that, for at least some cell types, there will be no significant delay between the fall in HoloTC concentration to below normal for the individual, and the rise in MMA and/or tHcy. This is consistent with Herbert (1987) where, in referring to the timing of a fall in HoloTC and the development of neutrophil hypersegmentation, he states that: “when one follows individuals from B-12 normality into the earliest stages of B-12 deficiency, one discovers that both fall almost together in the individual. The amount of B-12 on the B-12 delivery protein transcobalamin II falls sharply and, at almost the same time (probably fractionally later), DNA synthesis becomes subnormal in the granulocytes.” Even if not all cells are affected immediately, and if the MMA and/or tHcy are not yet measurably abnormally high, the metabolism for some cell types has been disturbed. Thus in this alternative model, almost immediately after the HoloTC production falls below normal for any reason, the *Depletion* stage ends and the *Metabolic Deficiency* stage commences.

### Dependence of timing of HoloTC response on cause of vitamin B_12_ deficiency

The magnitude and speed of any change in HoloTC concentration depends on the cause of the onset of vitamin B_12_ deficiency, as well as individual variables involved in the absorption, transport and utilization of vitamin B_12_. Three possible cases of disruption to the enterohepatic cycle are considered here: failure only after exhaustion of vitamin B_12_ liver store; instantaneous failure; gradual failure. The following discussion is based on this author’s hypothesis about how the response of total vitamin B_12_, HoloTC, tHcy and MMA depends on the cause of vitamin B_12_ deficiency. Human experimentation is needed to validate this model.

#### Failure only after exhaustion of vitamin B_12_ liver store

When there is no short-term disruption to the enterohepatic cycle, HoloTC concentration will be maintained until there is insufficient holohaptocorrin remaining to sustain HoloTC production. This case contains all stages of the general model, as shown in Fig. 5, where there is a significant *Depletion* period before the eventual failure of the enterohepatic cycle leads to the onset of *Metabolic Deficiency*. The total vitamin B_12_ falls, but HoloTC, MMA and tHcy remain normal, during the *Depletion* period. When the liver store of HoloHC is consumed, *Metabolic Deficiency* commences, with the HoloTC concentration falling and the MMA and tHcy increasing. This case applies where the failure is outside of the HoloTC feedback control loop (Fig. 4), for example when the vitamin B_12_ deficiency is caused by reduction in intake, or reduction in the utilization of the vitamin B_12_ in the cells because of a defect in the intracellular cobalamin metabolism. Another, very common, example of this case is food-cobalamin malabsorption where vitamin B_12_ bound to food protein cannot be released because of hypochlorhydria due to gastric atrophy (Carmel 1995; Andrès et al. 2004). This common cause of vitamin B_12_ deficiency in the elderly is not the same as pernicious anaemia because production of intrinsic factor is not affected; the patient is able to absorb unbound vitamin B_12_. Examples of such cases consistent with this alternative model, where vitamin B_12_ deficiency was likely to be caused by gradual loss of gastric acid production, were those whose subjects were elderly (Miller et al. 2006; Clarke et al. 2007; Schrempf et al. 2011; Palacios et al. 2013; Remacha et al. 2014).

#### Instantaneous failure

**Fig. 6:**
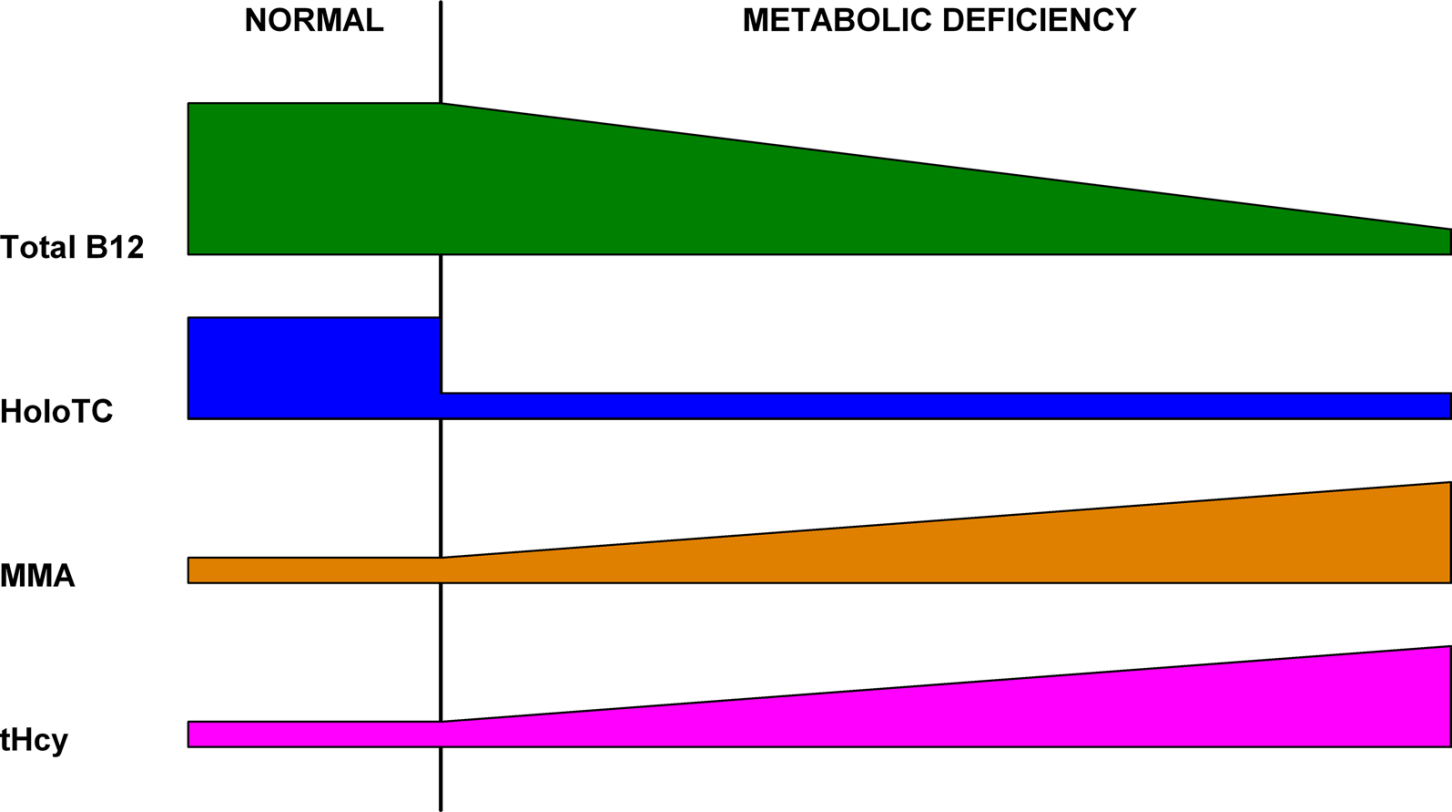
Instantaneous failure of enterohepatic recycling. Total B_12_, serum total vitamin B_12_ concentration; HoloTC, serum holotranscobalamin concentration; MMA, plasma methylmalonic acid concentration; tHcy, plasma total homocysteine concentration

If the production of HoloTC suddenly halts, because of an instantaneous failure of the enterohepatic cycle, for example as a result of the surgical removal of the parietal cells of the stomach, an extreme case of the proposed model will apply. As shown in Fig. 6, the sudden loss of HoloTC will cause an almost immediate metabolic disturbance and a rise in the concentration of MMA and/or HoloTC. Thus, there will be no *Depletion* period in this case, because MMA and/or tHcy will respond almost instantaneously to the fall in HoloTC concentration, and the *Metabolic Deficiency* stage commences almost immediately.

#### Gradual failure

**Fig. 7:**
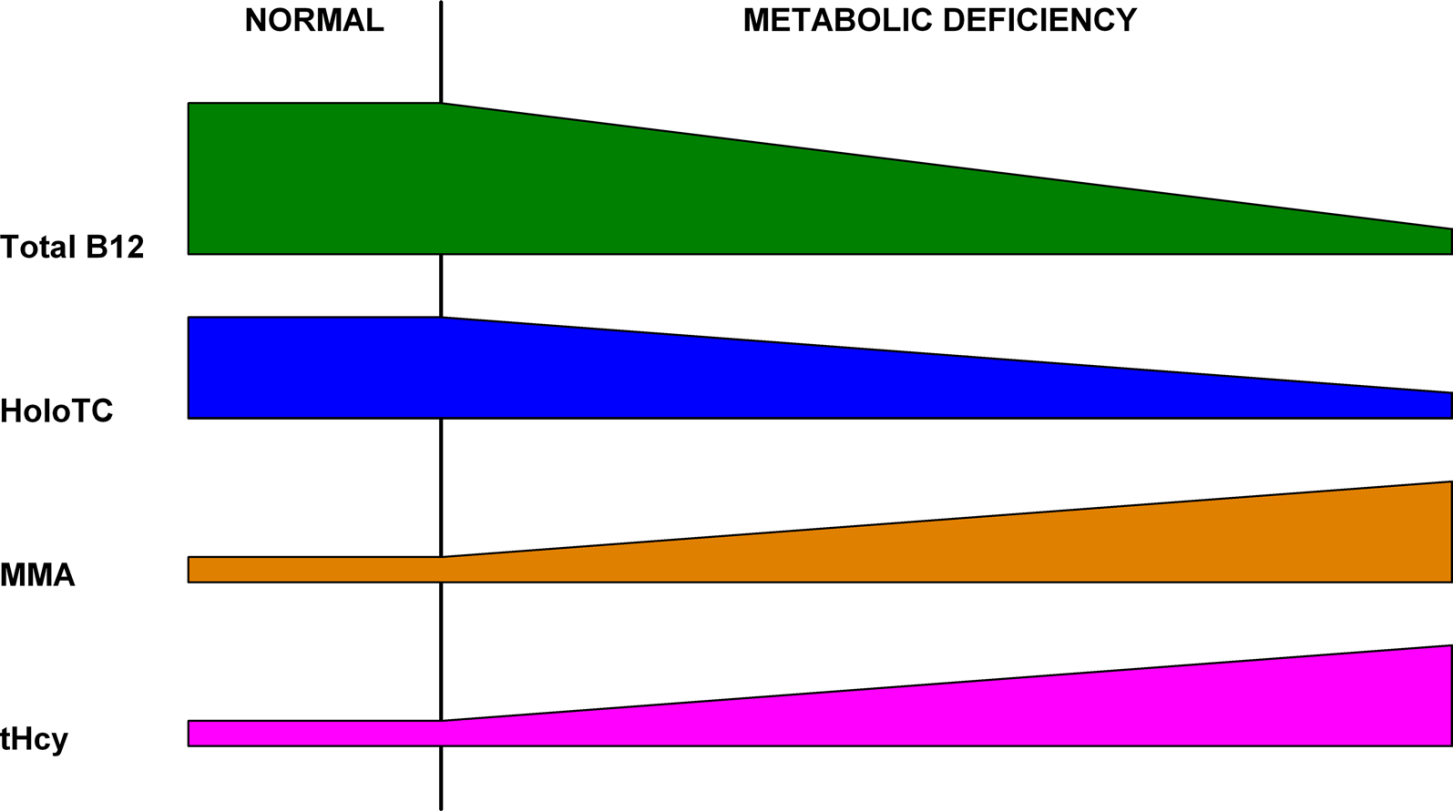
Gradual failure of enterohepatic recycling. Total B_12_, serum total vitamin B_12_ concentration; HoloTC, serum holotranscobalamin concentration; MMA, plasma methylmalonic acid concentration; tHcy, plasma total homocysteine concentration

Figure 7 illustrates the case where the production of HoloTC gradually fails, for example as a result of loss of parietal cell function due to the autoimmune disease pernicious anaemia. As for the case of instantaneous failure, there is no *Depletion* period, and the *Metabolic Deficiency* stage commences almost immediately, because MMA and/or tHcy will start to increase immediately after the HoloTC concentration falls below the normal level for the individual. The difference in this case is that the rate of change of the analytes will be slower than when the supply of intrinsic factor is suddenly stopped.

### Consequences of questioning Herbert’s model

Questioning of Herbert’s hypothesis that HoloTC is the most sensitive marker of *Early Negative Vitamin B*_12_*Balance*, or his model for the sequential stages in the development of vitamin B_12_ deficiency, is likely to raise strong objections.

Firstly, any model that is based on changes from individual normal analyte concentrations, instead of comparison with a universally accepted population normal, conflicts with the dogma that there must be a *gold*-*standard* test for vitamin B_12_ deficiency; either HoloTC or one a yet to be discovered.

Secondly, any model that requires a comparison of current analyte concentrations with a patient’s own normal levels would require proactive testing of all persons at risk, before the onset of any symptoms, to obtain their individual healthy baseline concentrations. Herbert himself suggested proactive testing, saying “serum holoTCIl should be measured every 5 y starting at age 55 because gradual loss of the ability to absorb vitamin B-12 occurs in everyone in a genetically determined, age-dependent pattern.” (Herbert 1994).

A potential alternative to obtaining a longitudinal record for individuals at risk is the use of an algorithm to combine the results of a one-off test of all four analytes into a single parameter. A mathematical model for this has been devised and described in detail by Fedosov (2010), and further tested and refined (Fedosov 2013; Fedosov et al. 2015; Brito et al. 2016). This author is cautious about this approach because, as with all other current methods, it might apply well to a population but not necessarily to an individual. This would need extensive testing on a wide range of subjects, for various causes of vitamin B_12_ deficiency, before it could be validated.

## Summary and conclusion

Axis-Shield and others, in promoting the commercialisation of the HoloTC immunoassay, have relied on Herbert’s erroneous hypothesis that “Ho1oTCII falls below the bottom of its normal range long before total serum vitamin B-12 … falls below the bottom of its normal range”, and his flawed model for the staged development of vitamin B_12_ deficiency (Herbert 1994).

Herbert’s model is flawed because it assumes that a normal minimum concentration for HoloTC may be universally defined. As with total vitamin B_12_, evidenced by the failure to find such a single cut-off value for HoloTC and instead the discovery of a wide *grey zone* for HoloTC, the range of normal for an individual is much smaller than for the population. As stated by Herbert (1994): “What is normal for one is not normal for another”.

Herbert’s hypothesis, that HoloTC will respond early to vitamin B_12_ depletion, before the onset of a clinical deficiency, is erroneous because it does not take into account how enterohepatic recycling regulates the HoloTC concentration. Where the cause of vitamin B_12_ deficiency does not disrupt the enterohepatic cycle, HoloTC will not be an early responder to a deficiency; there will be a *Depletion* period, in which total vitamin B_12_ falls, but HoloTC and the metabolites will only respond after the liver store is exhausted. When the cause of vitamin B_12_ deficiency does disrupt the enterohepatic cycle, the HoloTC will be an early responder but there will be no *Depletion* period; the metabolites will respond quickly when HoloTC falls below normal for the individual.

The HoloTC immunoassay cannot be used to measure vitamin B_12_ status any more reliably than total vitamin B_12_, or to predict the onset of a metabolic deficiency, because it is based on an erroneous hypothesis and a flawed model for the staged development of vitamin B_12_ deficiency.

## Additional files

10.1186/s40064-016-2252-z Figures 1 to 7, High-resolution images.
10.1186/s40064-016-2252-z Figures 1 to 7, High-resolution slides.
10.1186/s40064-016-2252-z Reference Data, tables.
