# Supplementary material for: Holotranscobalamin (HoloTC, Active-B12) and Herbert’s model for the development of vitamin B12 deficiency: a review and alternative hypothesis
Source: Springerplus. 2016 May 20;5(1):668. doi: 10.1186/s40064-016-2252-z (PMC4899389; doi:10.1186/s40064-016-2252-z)
Supplement: Supplementary file 1 — 10.1186/s40064-016-2252-z Figures 1 to 7, High-resolution images. [file 40064_2016_2252_MOESM1_ESM.pdf]

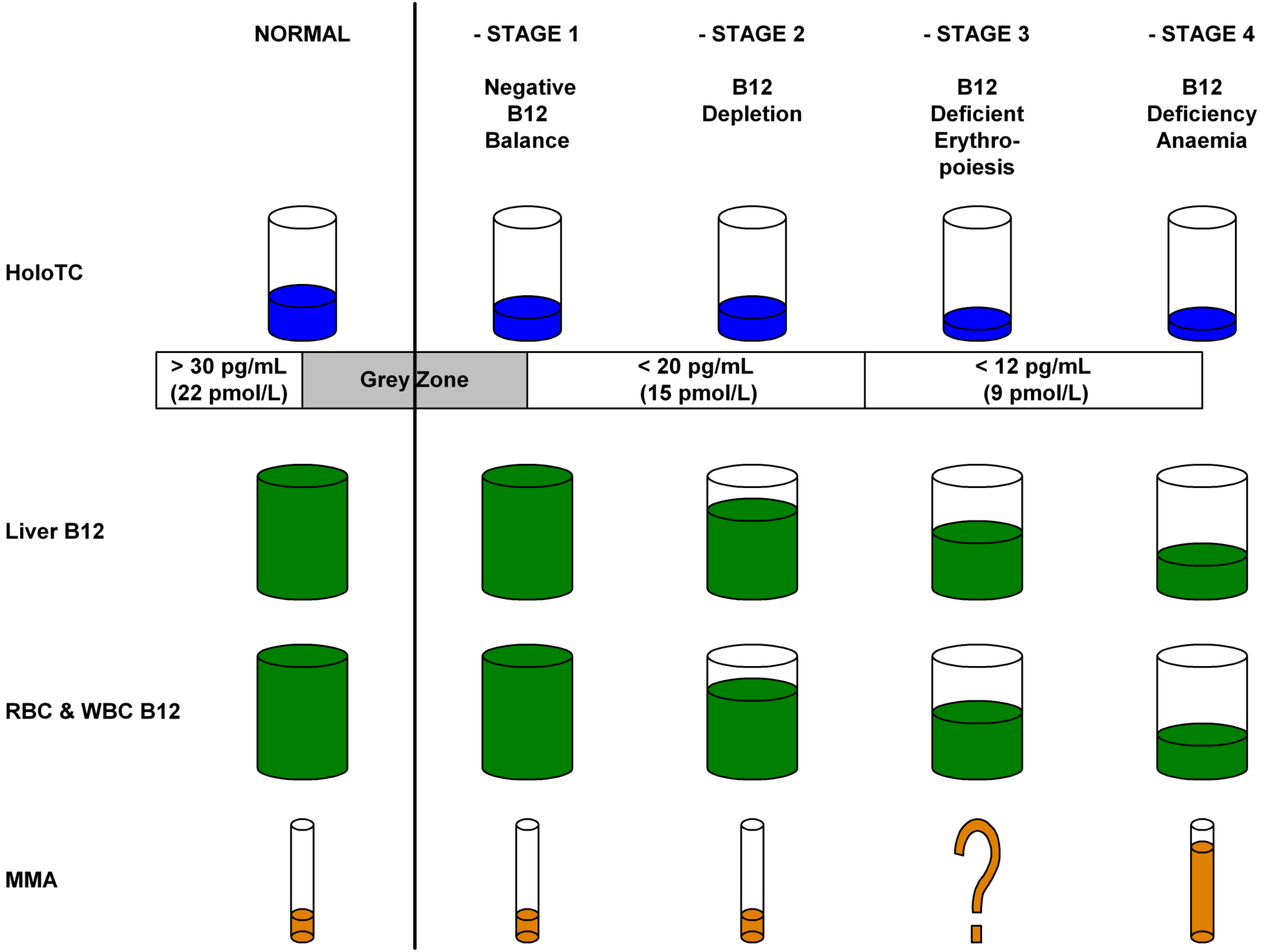
NORMAL
- STAGE 1
Negative B12 Balance
- STAGE 2
B12 Depletion
- STAGE 3
B12 Deficient Erythro-poiesis
- STAGE 4
B12 Deficiency Anaemia
HoloTC
> 30 pg/mL (22 pmol/L)
Grey Zone
< 20 pg/mL (15 pmol/L)
< 12 pg/mL (9 pmol/L)
Liver B12
RBC & WBC B12
MMA

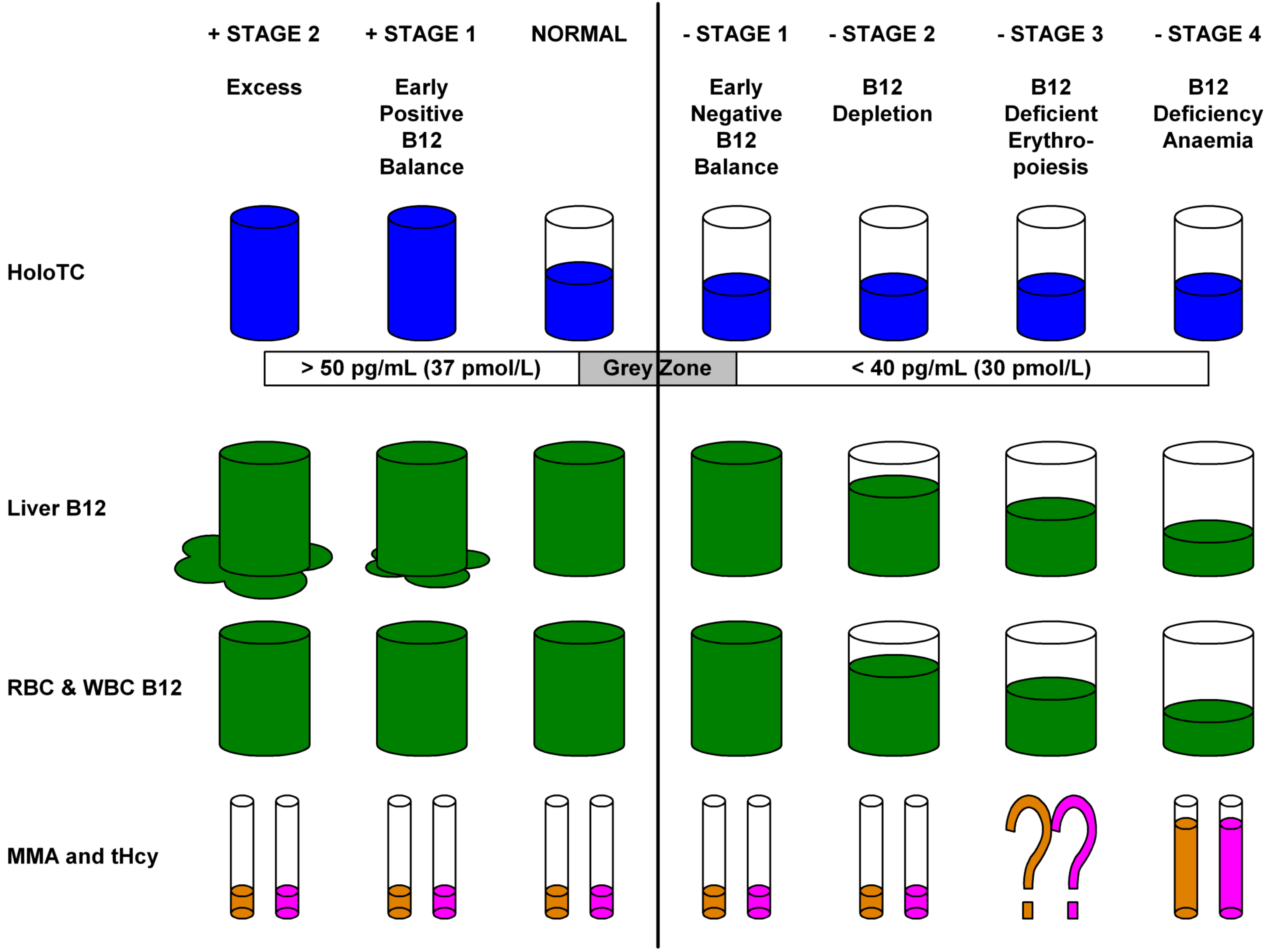
+ STAGE 2
+ STAGE 1
NORMAL
- STAGE 1
- STAGE 2
- STAGE 3
- STAGE 4
Excess
Early Positive B12 Balance
Early Negative B12 Balance
B12 Depletion
B12 Deficient Erythro-poiesis
B12 Deficiency Anaemia
HoloTC
> 50 pg/mL (37 pmol/L)
Grey Zone
< 40 pg/mL (30 pmol/L)
Liver B12
RBC & WBC B12
MMA and tHcy

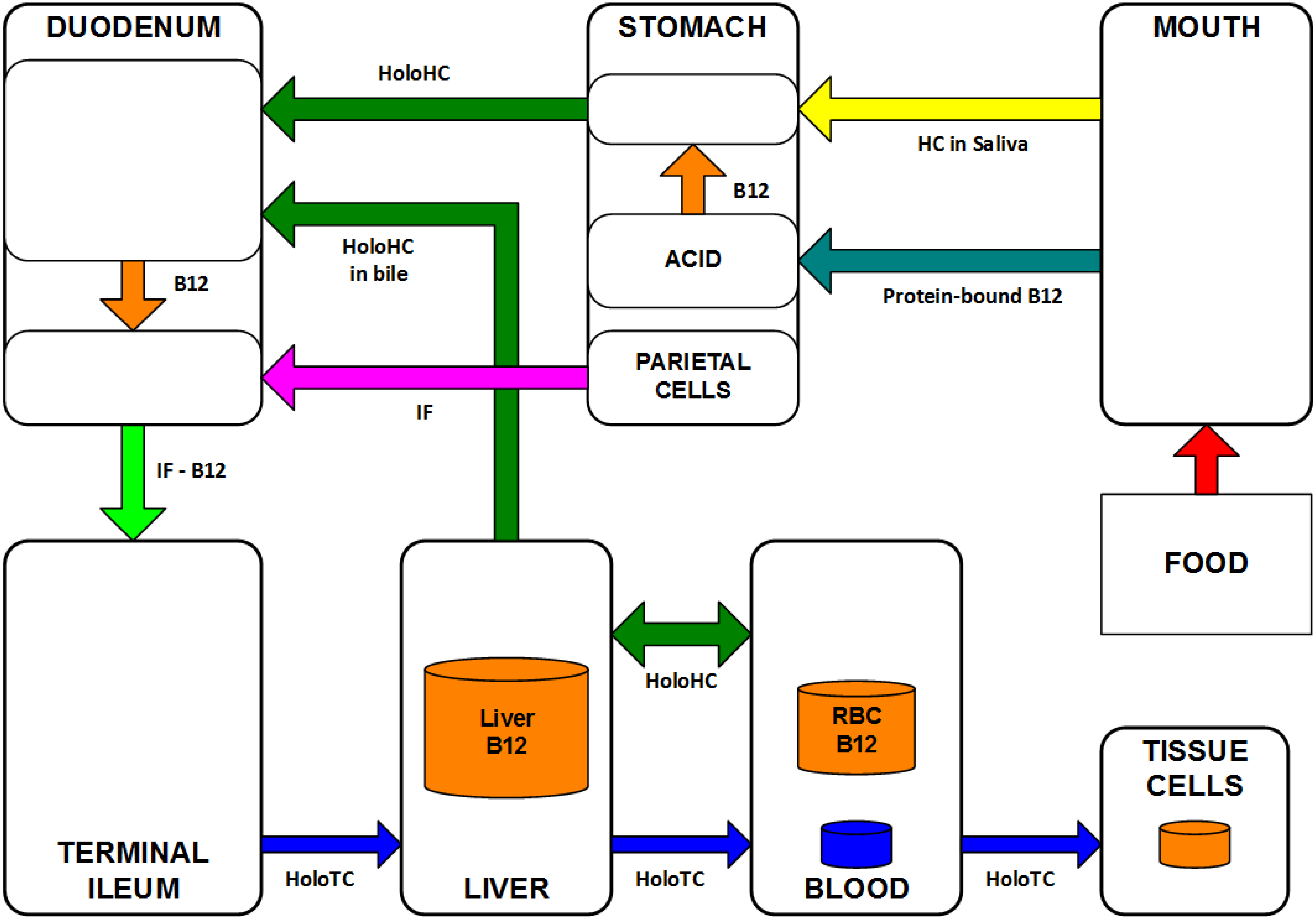
DUODENUM
STOMACH
MOUTH
HoloHC
HC in Saliva
B12
HoloHC
in bile
ACID
Protein-bound B12
B12
PARIETAL
CELLS
IF
IF - B12
FOOD
Liver
B12
HoloHC
RBC
B12
TISSUE
CELLS
TERMINAL
ILEUM
HoloTC
LIVER
HoloTC
BLOOD
HoloTC

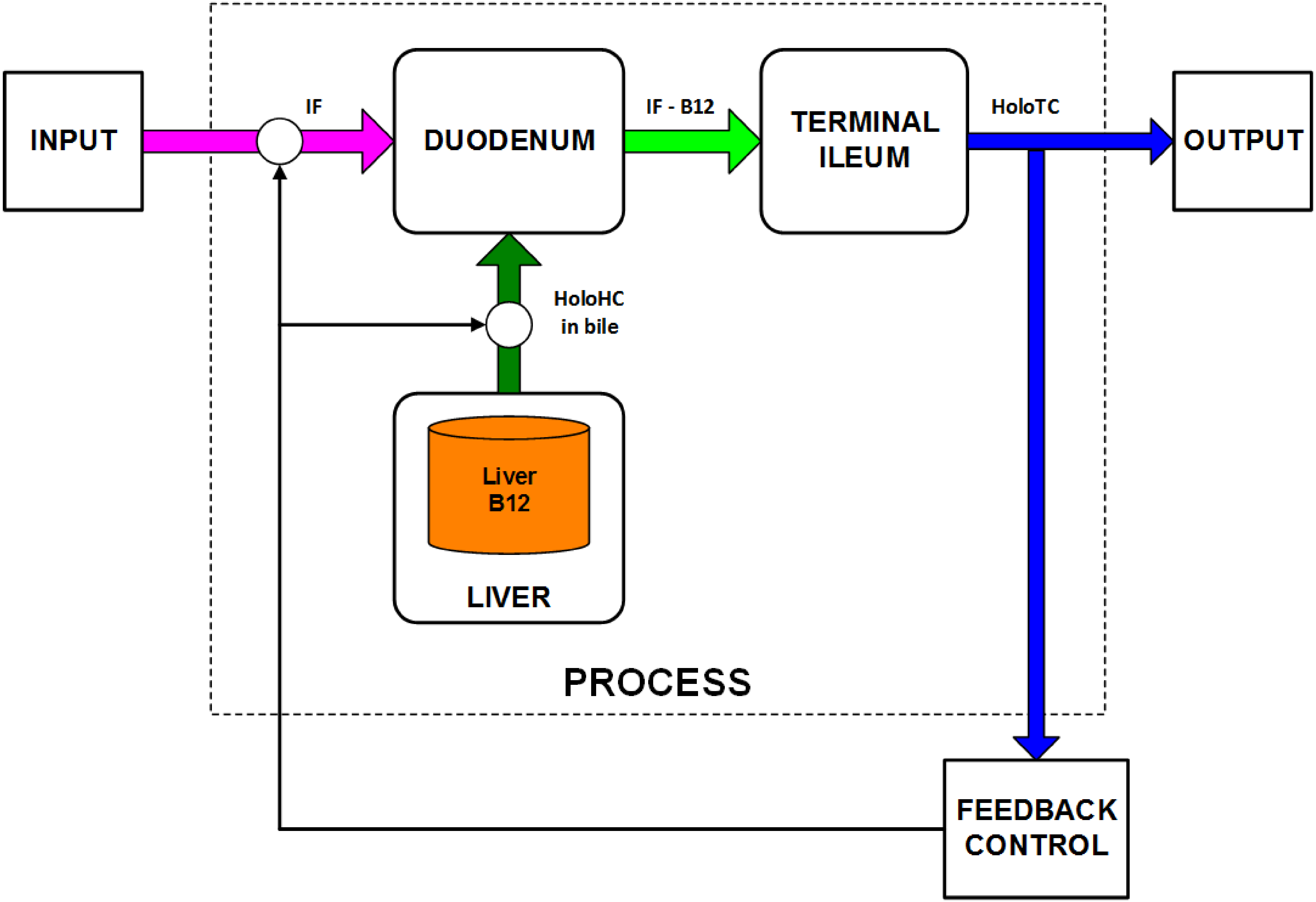
INPUT
IF
DUODENUM
IF - B12
TERMINAL ILEUM
HoloTC
OUTPUT
HoloHC in bile
Liver B12
LIVER
PROCESS
FEEDBACK CONTROL

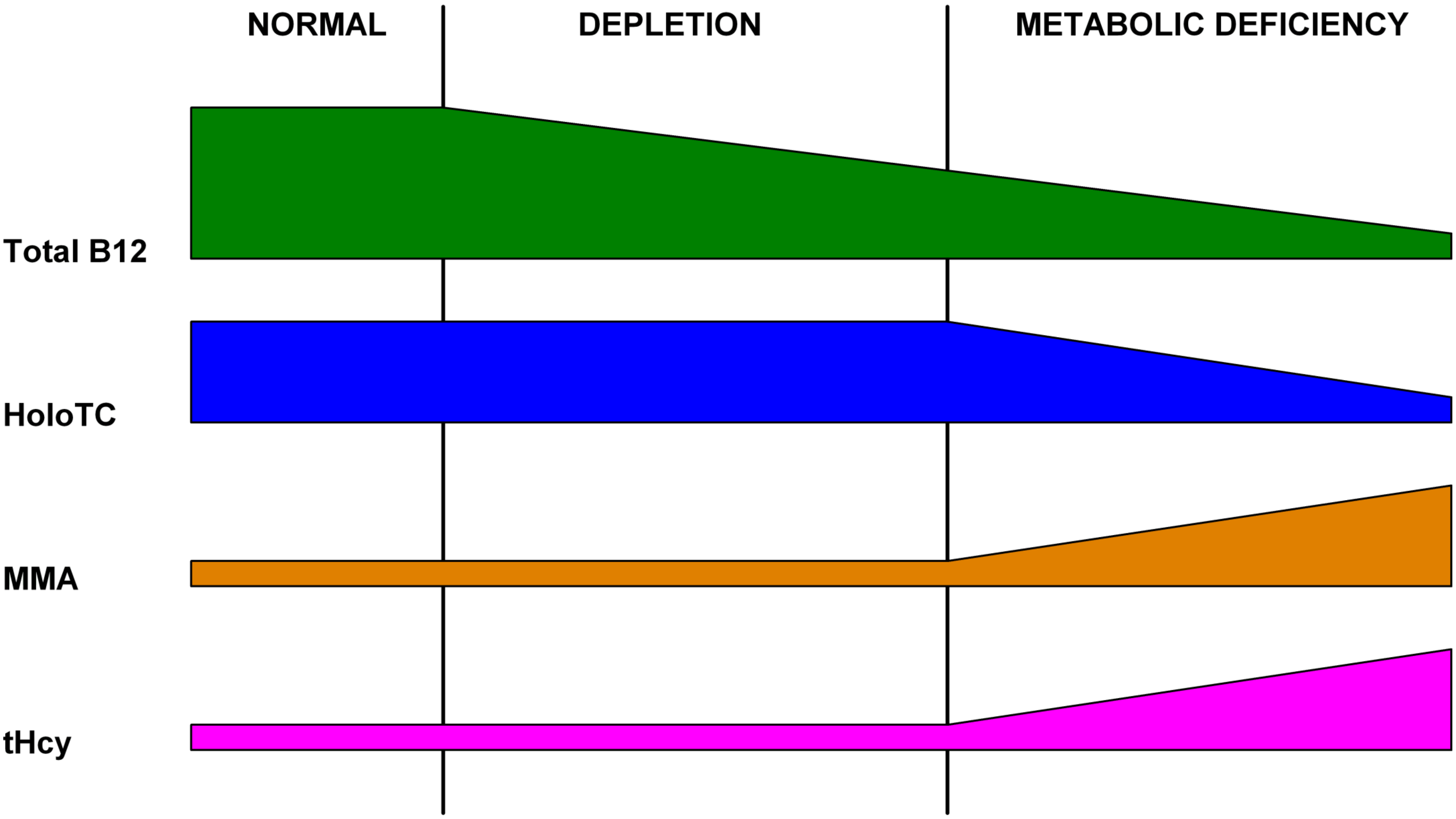
NORMAL
DEPLETION
METABOLIC DEFICIENCY
Total B12
HoloTC
MMA
tHcy

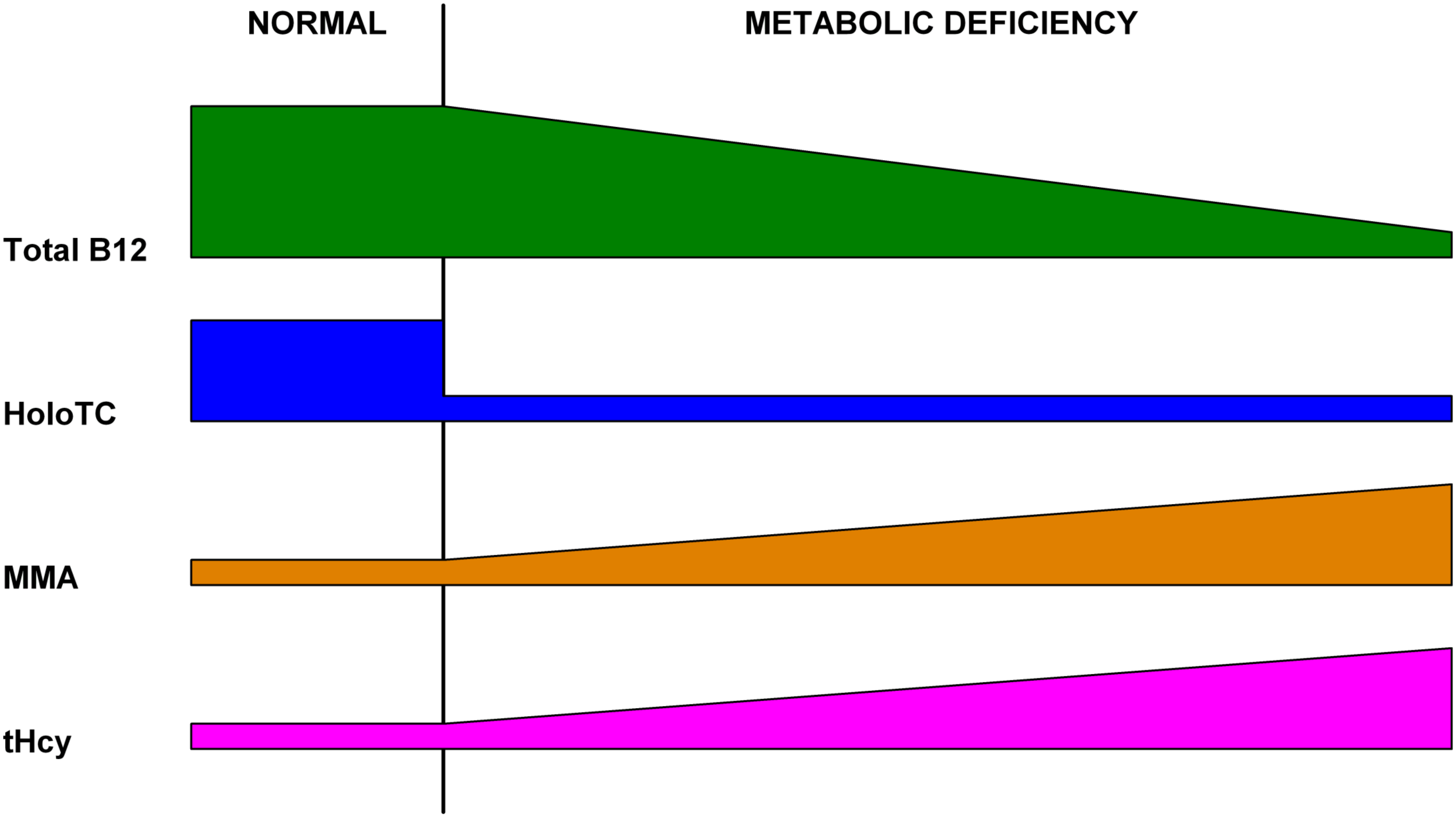
NORMAL
METABOLIC DEFICIENCY
Total B12
HoloTC
MMA
tHcy

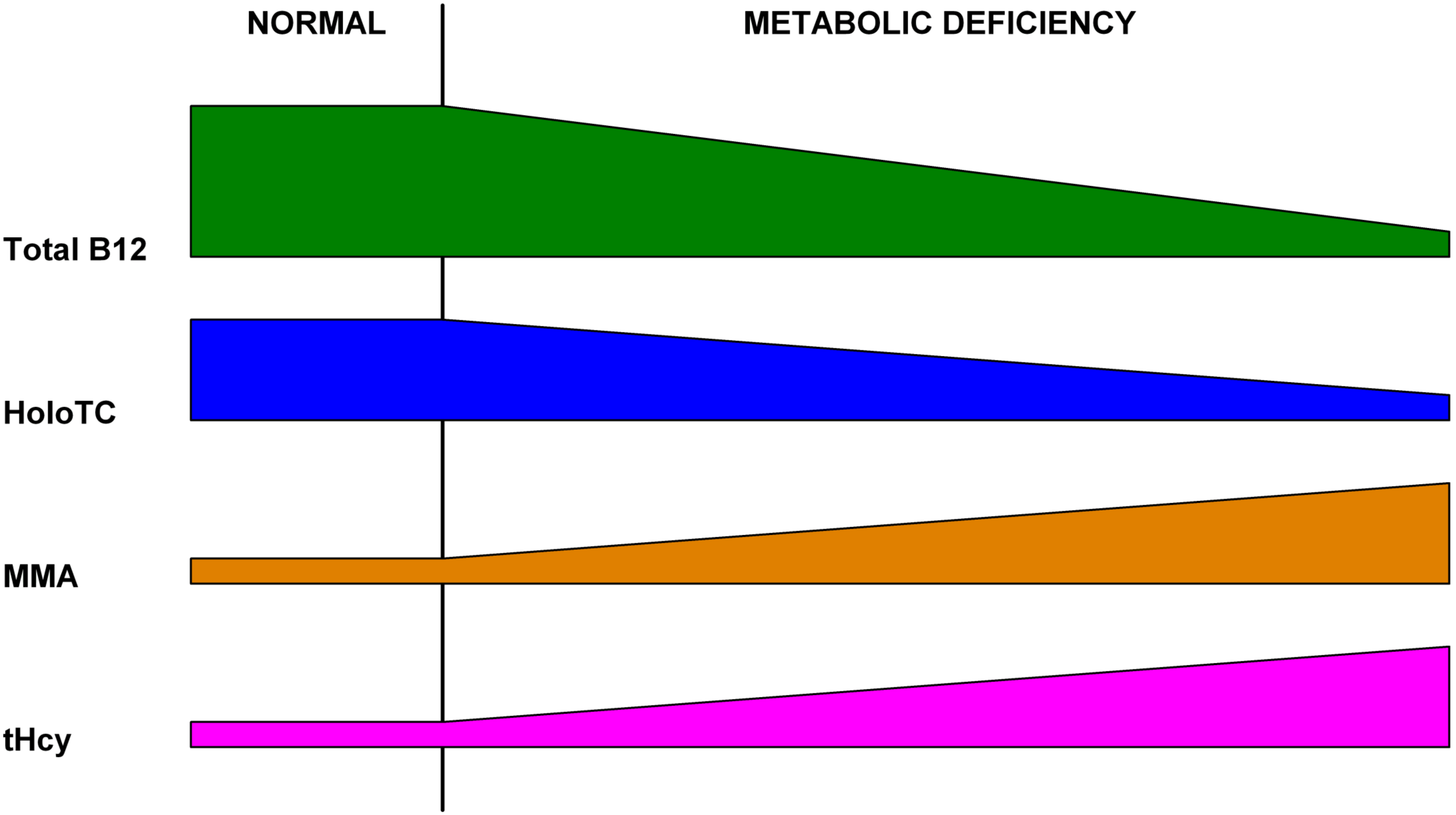
NORMAL
METABOLIC DEFICIENCY
Total B12
HoloTC
MMA
tHcy
