# Supplementary figures and images for: Holotranscobalamin (HoloTC, Active-B12) and Herbert’s model for the development of vitamin B12 deficiency: a review and alternative hypothesis
Source: Springerplus. 2016 May 20;5(1):668. doi: 10.1186/s40064-016-2252-z (PMC4899389; doi:10.1186/s40064-016-2252-z)

## Slide 1
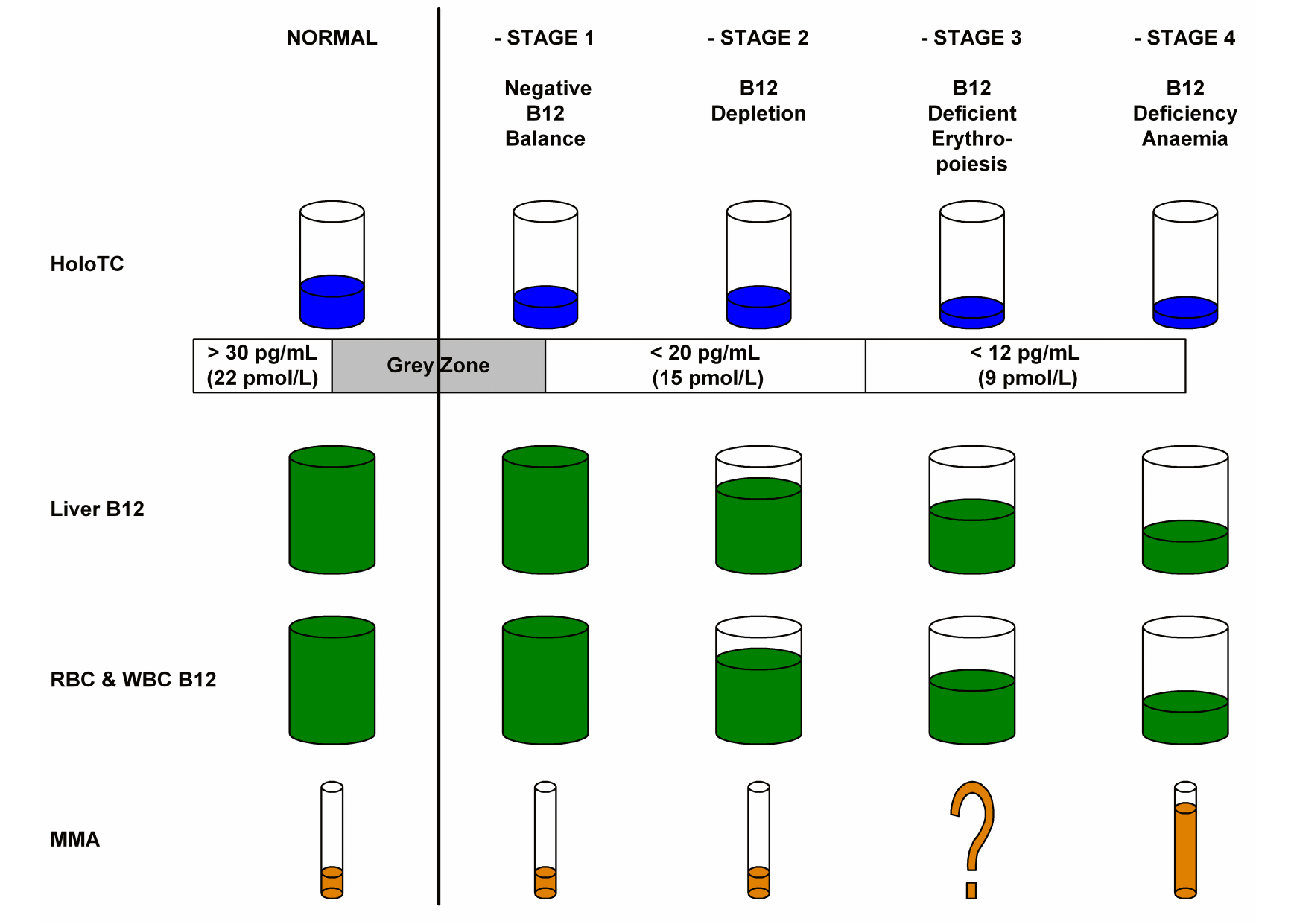

## Slide 2
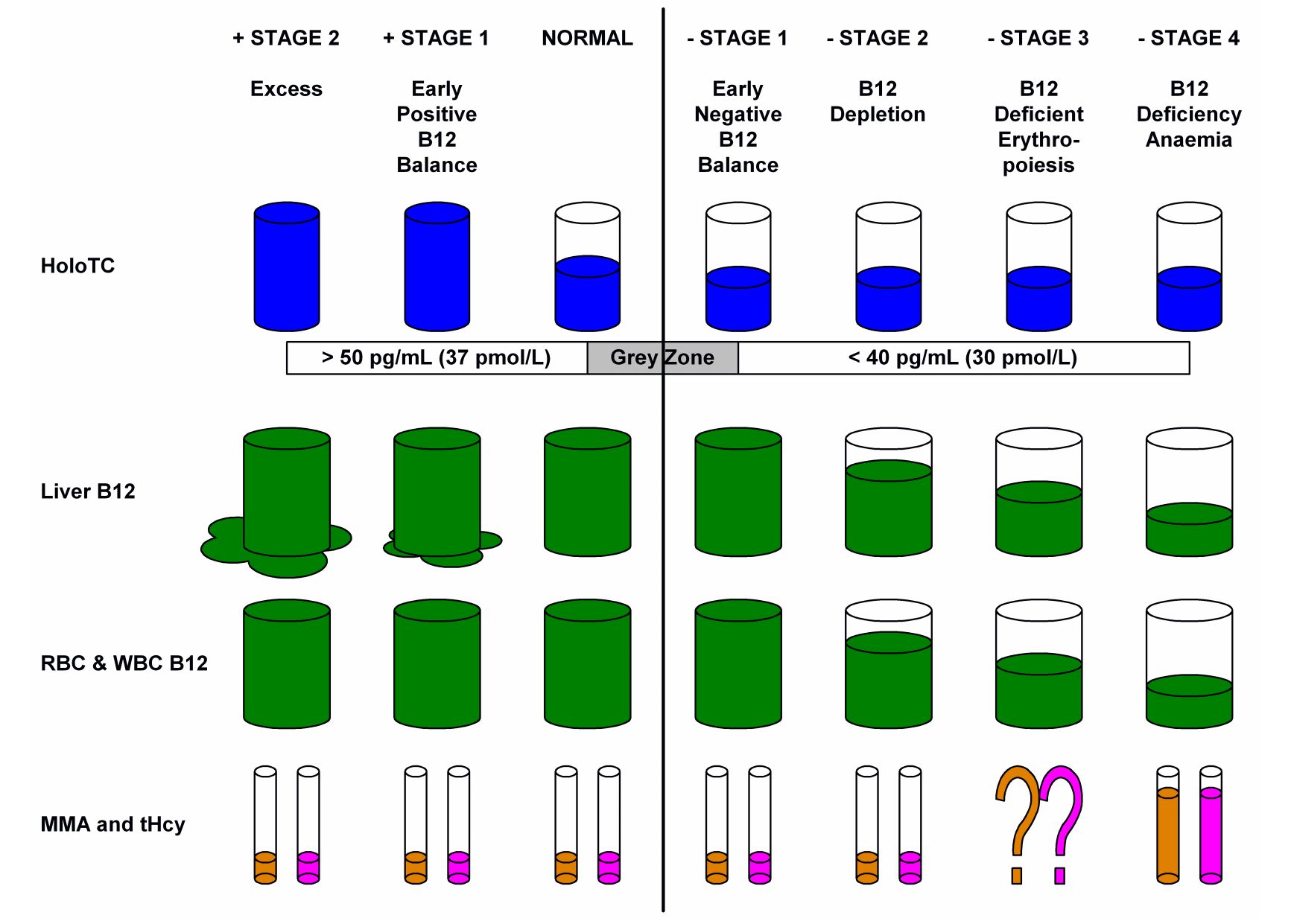

## Slide 3
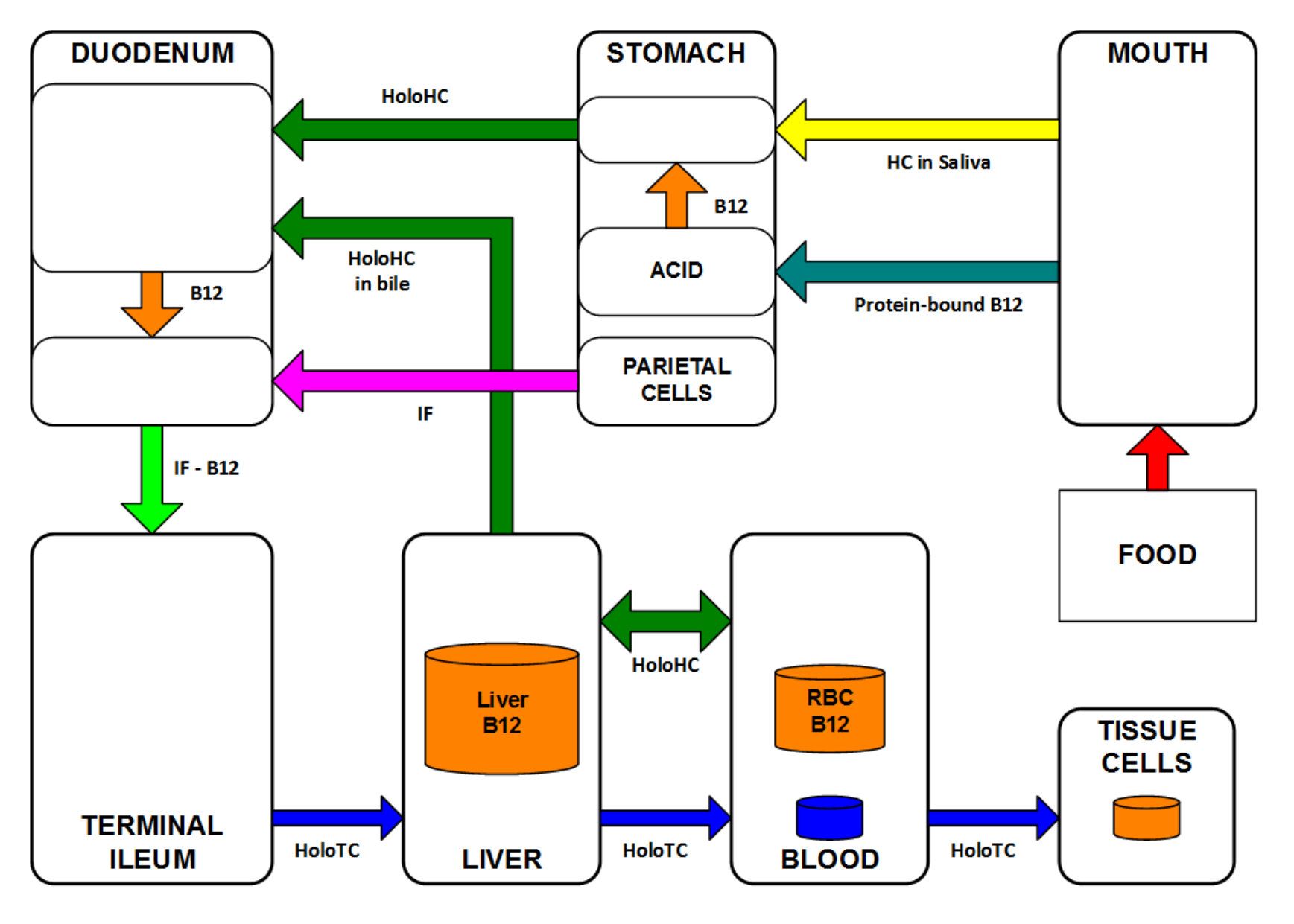

## Slide 4
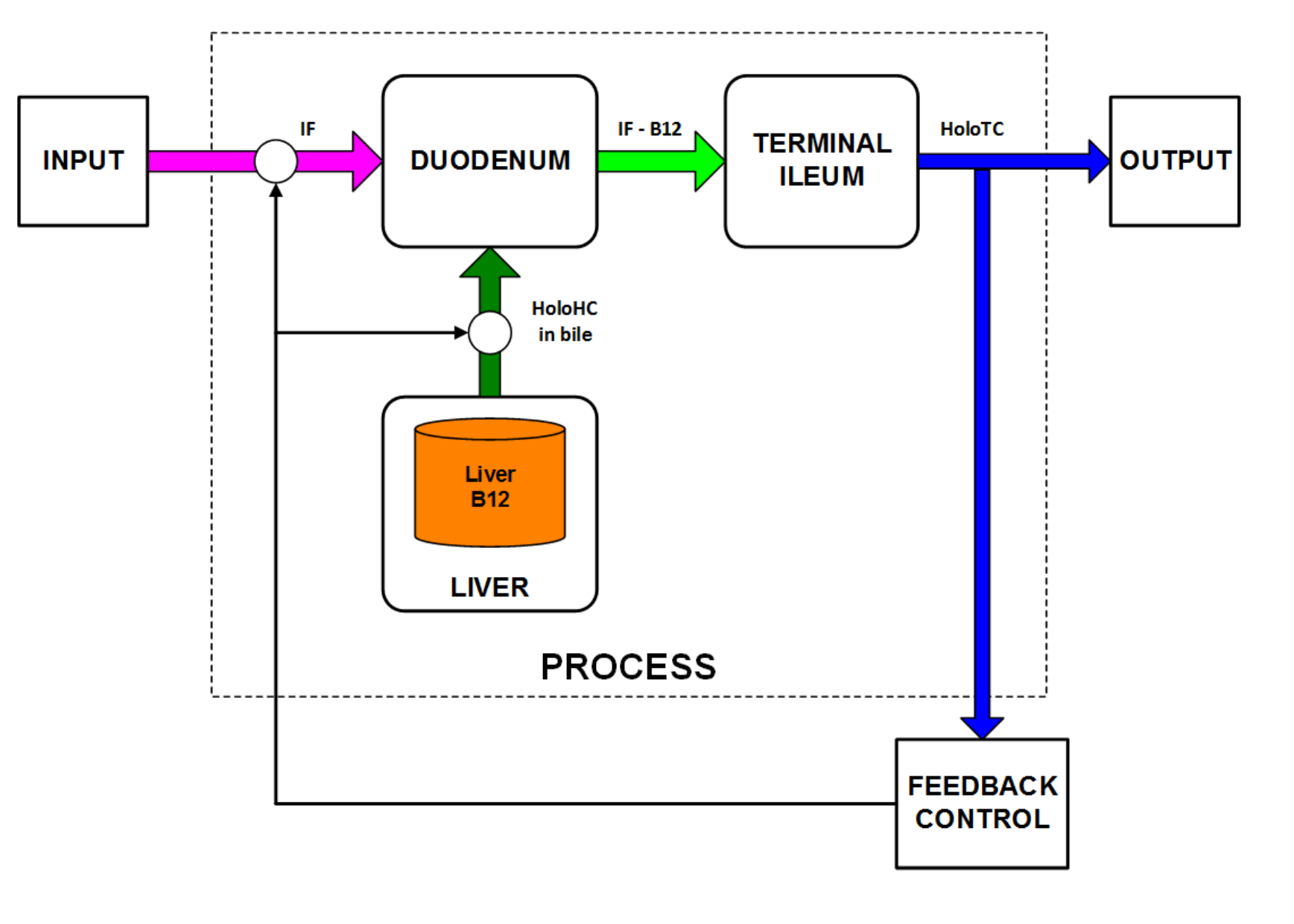

## Slide 5
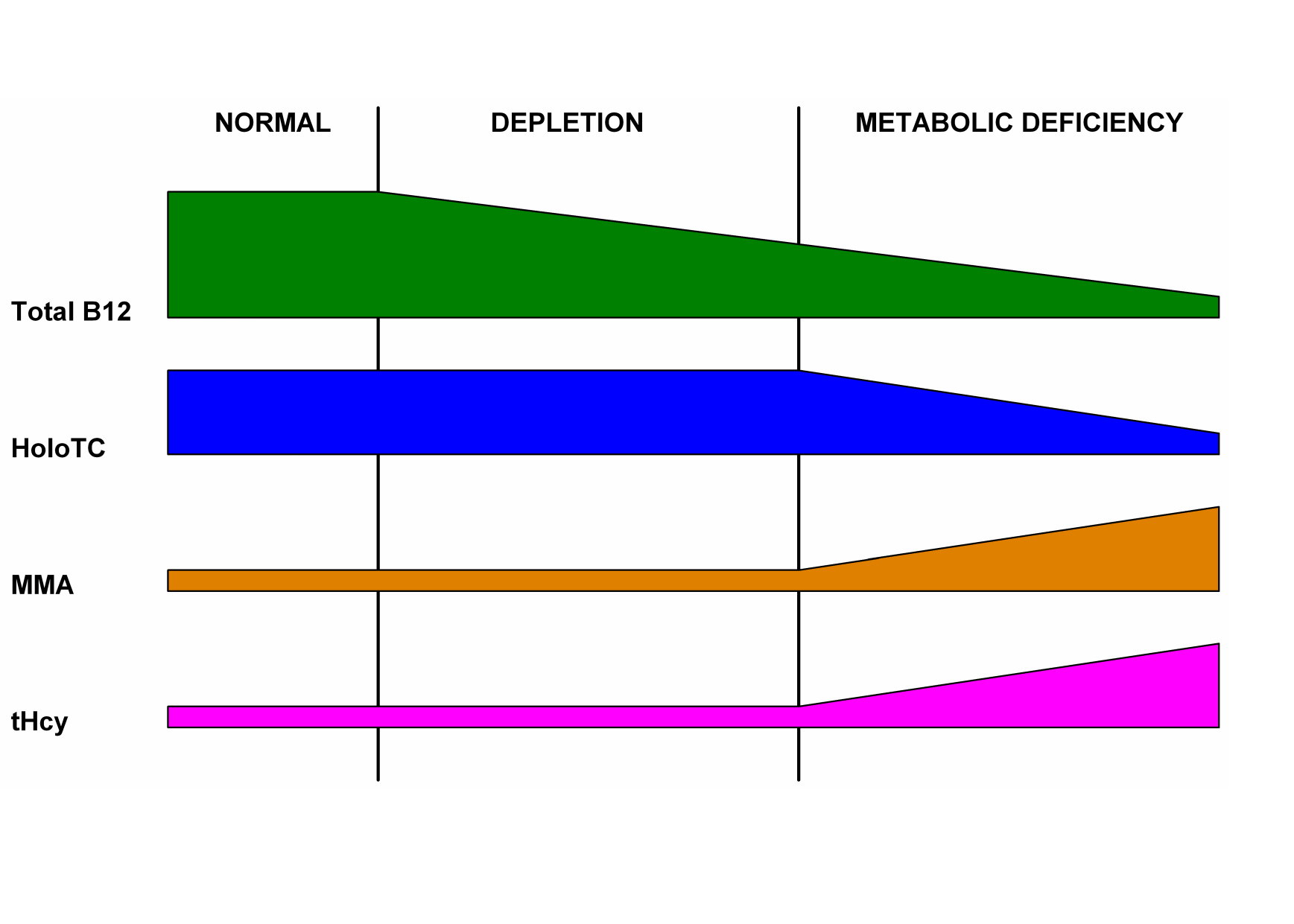

## Slide 6
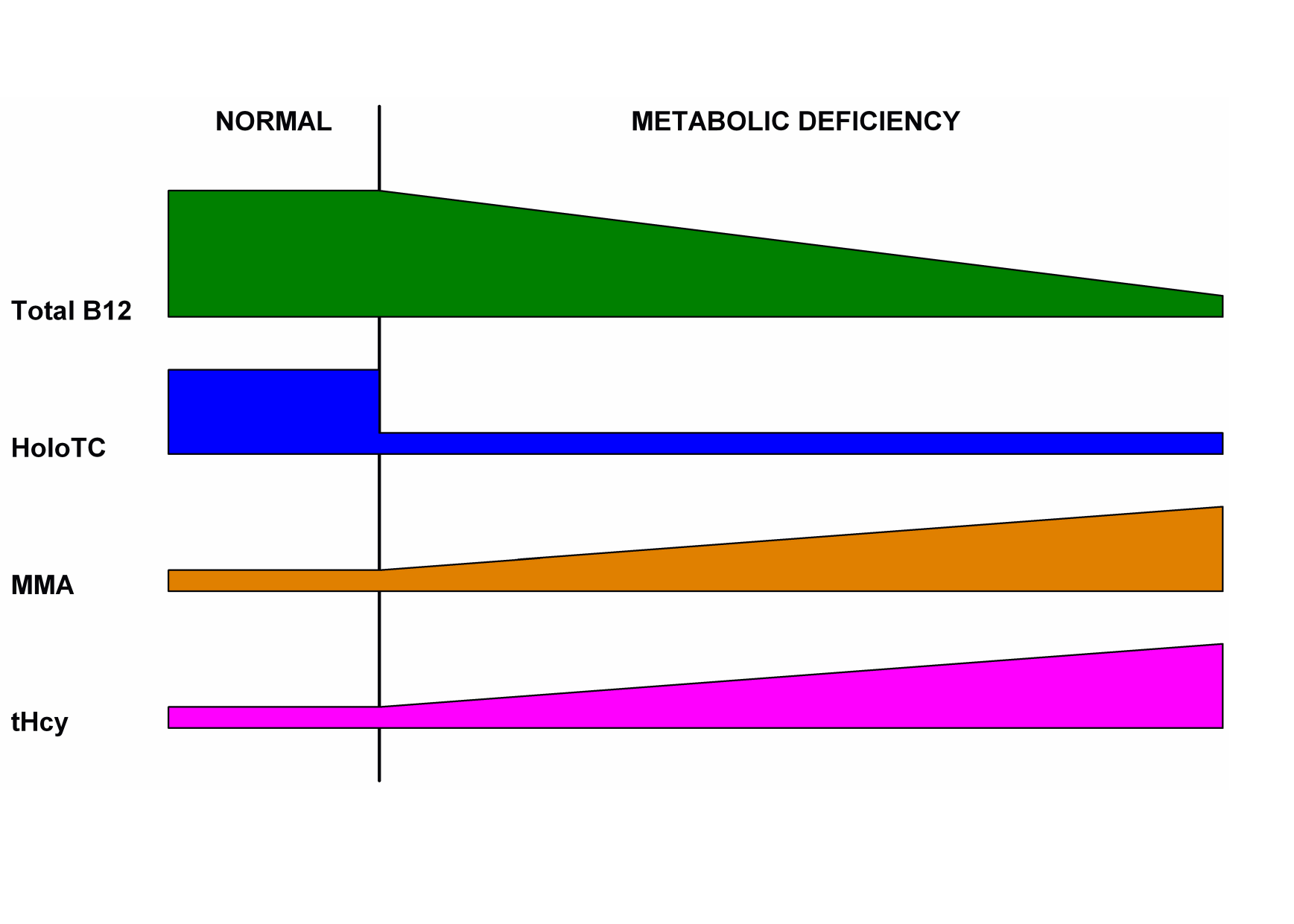

## Slide 7
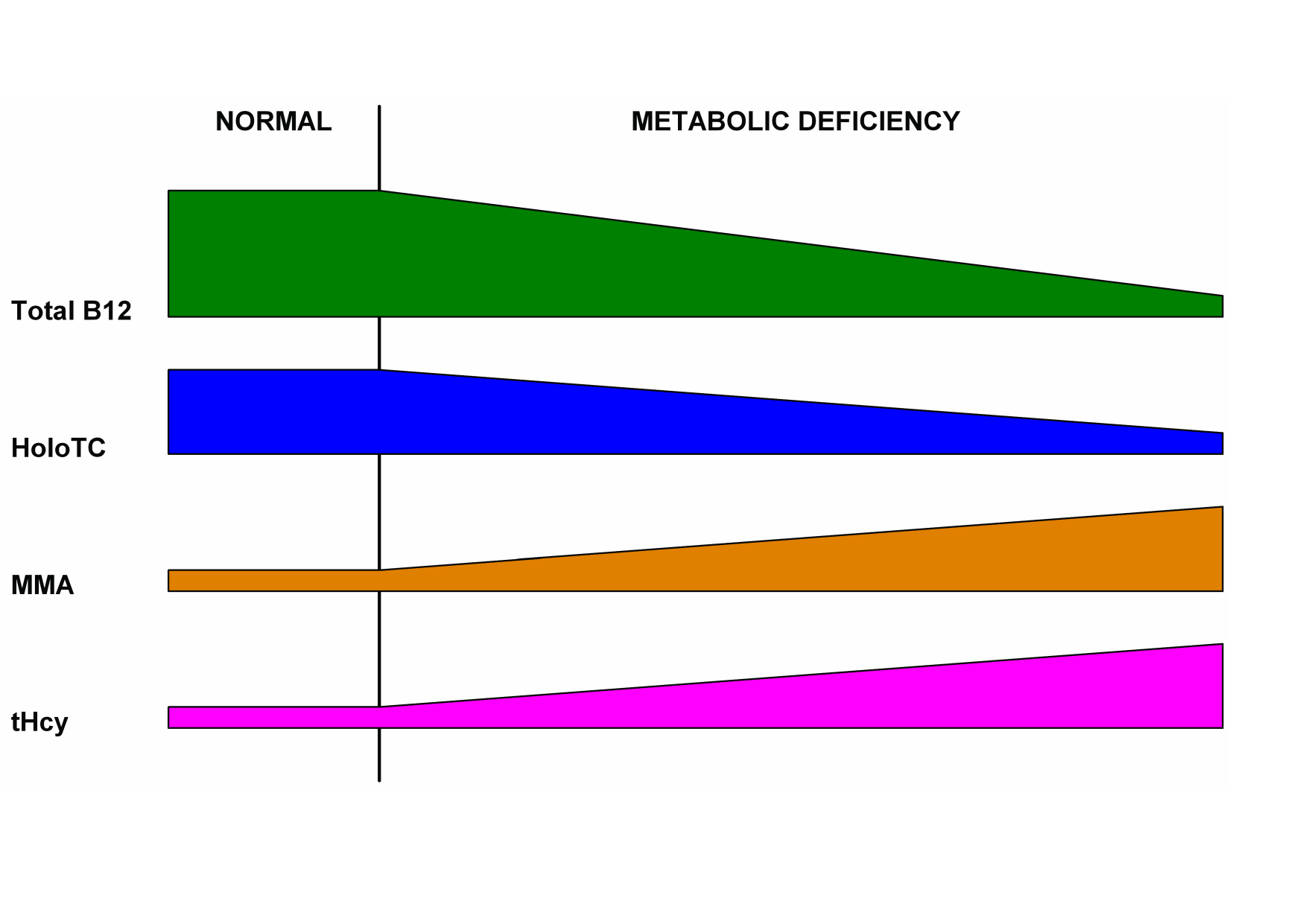

Supplement: Supplementary file 2 — 10.1186/s40064-016-2252-z Figures 1 to 7, High-resolution slides. [file 40064_2016_2252_MOESM2_ESM.pptx]
